# Downregulation of HNF4A enables transcriptomic reprogramming during the hepatic acute-phase response

**DOI:** 10.1038/s42003-024-06288-1

**Published:** 2024-05-16

**Authors:** Charlotte Ehle, Aishwarya Iyer-Bierhoff, Yunchen Wu, Shaojun Xing, Michael Kiehntopf, Alexander S. Mosig, Maren Godmann, Thorsten Heinzel

**Affiliations:** 1 Institute of Biochemistry and Biophysics, Center for Molecular Biomedicine, Friedrich Schiller University Jena, 07745 Jena, Germany; 2 Marshall Laboratory of Biomedical Engineering, Department of Pathogen Biology, Shenzhen University Medical School, Shenzhen University, Shenzhen, Guangdong 518060 China; 3 Department of Clinical Chemistry and Laboratory Diagnostics, Jena University Hospital, 07747 Jena, Germany; 4 Institute of Biochemistry II, Center for Sepsis Control and Care, Jena University Hospital, 07747 Jena, Germany

**Keywords:** Transcription, Hepatology

## Abstract

The hepatic acute-phase response is characterized by a massive upregulation of serum proteins, such as haptoglobin and serum amyloid A, at the expense of liver homeostatic functions. Although the transcription factor hepatocyte nuclear factor 4 alpha (HNF4A) has a well-established role in safeguarding liver function and its cistrome spans around 50% of liver-specific genes, its role in the acute-phase response has received little attention so far. We demonstrate that HNF4A binds to and represses acute-phase genes under basal conditions. The reprogramming of hepatic transcription during inflammation necessitates loss of HNF4A function to allow expression of acute-phase genes while liver homeostatic genes are repressed. In a pre-clinical liver organoid model overexpression of HNF4A maintained liver functionality in spite of inflammation-induced cell damage. Conversely, HNF4A overexpression potently impaired the acute-phase response by retaining chromatin at regulatory regions of acute-phase genes inaccessible to transcription. Taken together, our data extend the understanding of dual HNF4A action as transcriptional activator and repressor, establishing HNF4A as gatekeeper for the hepatic acute-phase response.

## Abstract (web summary)

Ehle et al. show that downregulation of the liver-identity factor HNF4A facilitates inflammation-associated acute phase gene expression at the expense of homeostatic hepatic functions, to promote the acute phase response.

## Introduction

Liver-enriched transcription factors (LETFs) including FOXA2, GATA6, HNF1A, LRH1, FXRA, PXR, C/EBPA and HNF4A cooperatively shape active enhancer-promoter landscapes to establish hepatocyte-specific gene expression^1–4^. LETFs thereby enforce a transcriptional program that enables homeostatic liver functions including detoxification, gluconeogenesis, synthesis of steroids, cholesterol, bile acids, glycogen, urea, and regulation of lipid metabolism. Moreover, LETFs co-regulate their own expression in autoregulatory circuits where the hepatocyte nuclear factor 4 alpha (HNF4A) has a pivotal role to sustain the expression of the entire network of LETFs and thereby to maintain the homeostatic liver transcriptome^5,6^.

HNF4A is an orphan nuclear receptor and is classified into two functionally distinct isoform types - P1 and P2 – which are each transcribed from their own promoter^7,8^. The P1 isoform variants (HNF4A1-6) maintain the hepatic-identity expression profile and are predominantly expressed in the adult liver, whereas the P2-derived variants (HNF4A7-12) are associated with embryonic liver development and disease states^9,10^. HNF4A P1 binds to enhancers and promoters of > 50% of liver-specific genes^6^, recruiting co-activators (e.g. P300, TET3) to establish active chromatin states associated with epigenetic modifications such as histone H3K4me1, H3K27ac and 5-hydroxymethylation of cytosines^1,3,11^. In addition, HNF4A directly loads RNA Polymerase II (Pol II) to promoters^12^ and possesses pioneering activity to open previously inaccessible chromatin^13^. While HNF4A DNA binding has been primarily associated with transactivation, recent studies suggest a repressive function mediated by its C-terminal repressive F-domain^14–16^ and recruitment of co-repressors^17^.

The acute-phase response (APR) constitutes the hepatic response to acute infection, injury and pro-inflammatory signals such as interleukin (IL)-6, IL1β or tumor necrosis factor alpha (TNFα) which enable the massive induction of serum proteins (acute-phase proteins - APPs) with a concomitant suppression of homeostatic liver function^18,19^. APPs like serum amyloid A (SAA), haptoglobin (HP), fibrinogens, alpha-1 antitrypsin (AAT) or C-reactive protein (CRP) support the systemic immune response and are crucial to eliminate pathogens and to limit tissue damage. Acute-phase (AP) gene expression is mainly induced by NF-κB p65, STAT3 and C/EBPB^20–22^, acting in a synergistic or antagonistic manner^23,24^. The role of LETFs within transcriptomic reprogramming during the APR is however less well investigated^25–27^.

Reduced abundancy and activity of LETFs and predominantly HNF4A P1 is strongly associated with liver dysfunction and pathologies like non-alcoholic fatty liver disease (NAFLD), hepatitis, cirrhosis or acute liver failure^28–30^. In fact, transient loss of hepatocyte identity by impairment of HNF4A was proposed as protective mechanism to reallocate cellular resources to stress responses and to stimulate hepatocyte regeneration^31–33^. In the context of the hepatic acute-phase response, however, little attention has been paid to the contribution of LETFs and especially HNF4A in regulating transcriptional adaption to inflammation.

We hypothesized that the unique feature of HNF4A to act both as transactivator and transrepressor puts it in a critical role to control transcriptome reprogramming in response to inflammation, or in general stress responses. Transcriptome profiling of lipopolysaccharide (LPS)-treated hepatocyte derived HepaRG cells reaffirmed HNF4A as an important regulatory hub. In unstimulated conditions, HNF4A chromatin immunoprecipitation (ChIP) in combination with formaldehyde-assisted isolation of regulatory elements (FAIRE)-qPCR revealed that occupancy of HNF4A at regulatory regions of acute-phase genes was associated with an inaccessible chromatin state. Conversely, overexpression of HNF4A interfered with reprogramming towards an inflammatory expression profile and prevented the suppression of liver homeostatic genes. These findings add to the emerging idea of transiently suppressed liver function to mount cellular stress-coping mechanisms such as the hepatic acute-phase response.

## Results

### HNF4A is a central node in hepatic transcriptome changes during the APR

**Fig. 1:**
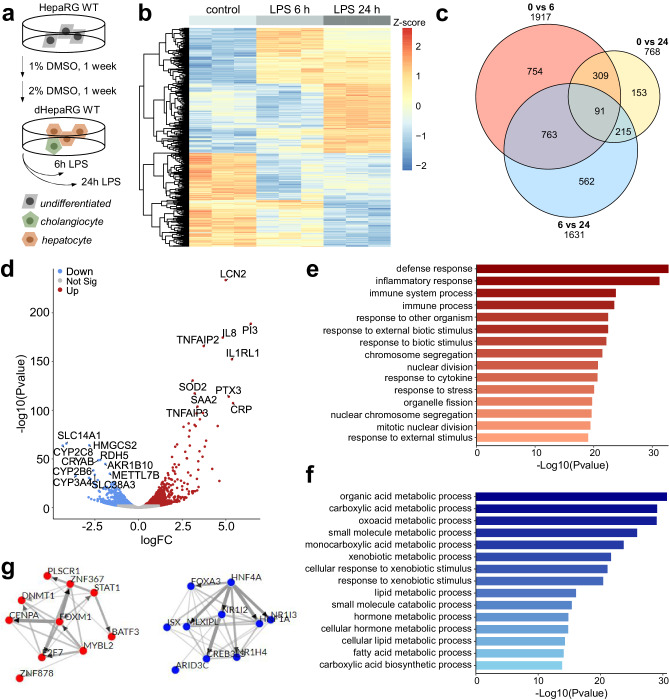
Transcriptome reprogramming during the acute phase response involves liver-specific transcription factors. **a** Scheme showing differentiation and treatment regimen for the dHepaRG cell line. **b** Heatmap depicting the Z-score of differentially expressed genes (DEGs) comparing 0 vs 24 h LPS set. **c** Venn diagram for overlap of DEGs comparing 0 vs 6 h (red), 0 vs 24 h (yellow) and 6 h vs 24 h (blue). **d** Volcano plot of DEGs (0 vs 24 h LPS). **e**, **f** Top 15 gene ontologies (KEGG, biological process) of upregulated (**e**) or downregulated (**f**) gene sets for the comparison 0 vs 24 h. **g** ChEA3 TF-TF co-regulatory network analysis of up- (red) and downregulated (blue) set of DEGs.

In order to assess the hepatic gene expression changes during the APR, we performed transcriptomic analysis of differentiated HepaRG (dHepaRG) cells treated with LPS for 6 h and 24 h. (Fig. 1a). RNA-Seq analysis revealed a differential regulation of 1917 genes at 6 h (888 up, 1107 down), 400 of which were significantly altered after 24 h (Fig. 1b, c). Compared to control conditions, 768 genes were differentially regulated at 24 h (462 up, 353 down). Among the ontologies that were commonly enriched in the upregulated gene set at 6 h or 24 h were pathways involved in immune and defense response (Fig. 1e). Top genes showing the highest fold change were prominent acute-phase genes such as *LCN2* (lipocalin 2, log_2_FC = 4.99), *CRP* (C-reactive protein, log_2_FC = 5.41) and *SAA2* (serum amyloid A-2, log_2_FC = 3.21) (Fig. 1d). A list for the expression data of all 30 designated acute-phase genes can be found in Table S1.

Gene ontology analysis of the downregulated gene set revealed that processes involved in crucial liver function such as xenobiotics, bile acid, lipid metabolism, and synthesizing processes linked to amino acids or hormones were markedly impaired upon LPS treatment (Fig. 1f). These processes were already downregulated after 6 h and remained repressed after 24 h. 1631 differentially expressed genes (DEGs) were detected between the 24 h and 6 h timepoint, revealing a dynamic activation of immune response pathways which peaked at 6 h but was already reduced after 24 h (Fig. S1a, b). Additionally, ontologies related to cell division and proliferation were enriched at the 24 h timepoint, implying a progressive loss of differentiated, functional hepatocytes. These results show that transcriptome changes are induced rapidly to activate the hepatic immune response, with a concomitant repression and sustained attenuation of metabolic regulatory pathways (Fig. S1c) that culminates in hepatic de-differentiation.

To gain further understanding which transcriptional regulators were involved in these reprogramming processes, we utilized the ChEA3 tool for transcription factor enrichment analysis^34^. This tool integrates multiple libraries of transcription factor (TF) target genes, including ChIP-Seq (ENCODE, ReMap, individual sources) and co-expression data derived from RNA-Seq (GTEx, ARCHS4) and thus predicts TFs that are associated with regulation of the input gene set. In addition, a weighted gene co-expression network analysis is performed and visualized as a global or local TF-TF co-regulatory network based on the top results from the ChEA3 prediction. Liver-specific nuclear receptors like NR1I2 (PXR, pregnane X receptor), NR1H4 (BAR, bile acid receptor), NR1I3 (CAR, constitutive androstane receptor) and HNF1A (hepatic nuclear factor 1 alpha) were enriched in pathways that were downregulated after 6 h and 24 h (Fig. 1g). Most notably, the transcription factor HNF4A was depicted as the central node within the local TF-TF co-regulatory network, underscoring that HNF4A is a major factor regulating the differentially expression genes during the APR.

### Inflammatory signals rapidly repress HNF4A transcription

**Fig. 2:**
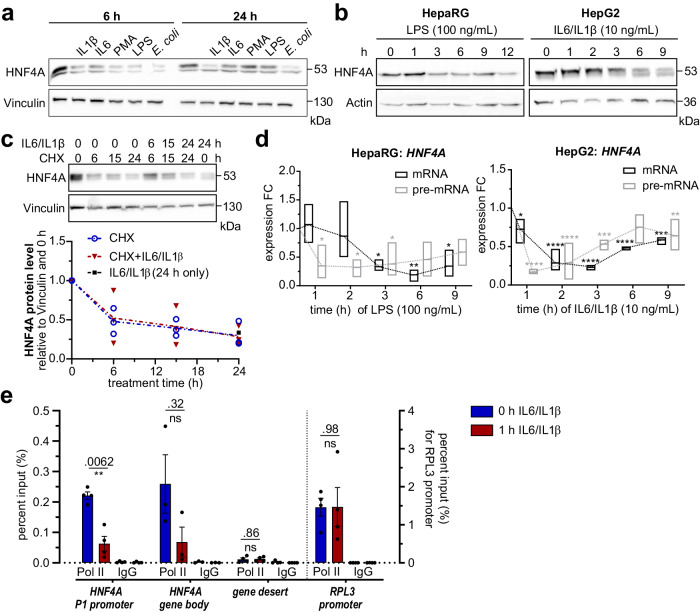
Transcriptional suppression of HNF4A by inflammatory stimuli. **a** Western blot of dHepaRG treated for 6 h or 24 h with different pro-inflammatory stimuli. **b** Time course of HNF4A protein loss in LPS-treated HepaRG and cytokine-treated HepG2. **c** CHX chase experiment for HepG2 treated with CHX alone (10 µM) or in combination with cytokines (10 ng/mL) for 6 h, 15 h or 24 h respectively. Densitometric analysis from *n* = 3 independent biological replicates is shown below. **d** RT-qPCR for pre-mRNA and mRNA levels of *HNF4A* P1 in LPS-treated HepaRG or cytokine-treated HepG2 for 1 h-9 h (*n* = 3, data displayed from min-max, line at mean, 2-way ANOVA post-hoc: Holm-Sidak test, stars indicate significance compared to timepoint 0 h). **e** ChIP-qPCR showing RNA-Pol II occupancy at the indicated gene regions in HepG2 treated for 1 h with 10 ng/mL IL6/IL1b (*n* = 4, data are expressed as mean ± SEM, 2-way ANOVA post-hoc: Holm-Sidak). **a**–**c** Western blots show whole cell protein extracts. Significance levels *< 0.05; **< 0.01; ***< 0.001; ****< 0.0001.

To investigate how HNF4A expression is regulated during the APR, we treated dHepaRG cells with pro-inflammatory stimuli and monitored HNF4A levels by western blotting. In response to IL6 and IL1β or endotoxin stimulation (LPS or heat-inactivated *E. coli*), HNF4A protein levels were time-dependently downregulated (Fig. 2a). This effect was prominent as early as 3–6 h after treatment (Fig. 2b). Cycloheximide (CHX) chase assay indicated that HNF4A has a half-life of approximately 6 h in both dHepaRG and HepG2 cell lines (Fig. 2c). Cytokine treatment in addition to CHX did not further reduce HNF4A protein levels, indicating that the inflammation-induced loss of HNF4A is not resulting from accelerated proteasomal degradation but rather a transcriptional effect.

To this end mRNA and pre-mRNA levels of *HNF4A* P1 transcripts were monitored by RT-qPCR (see Table S2). Transcript variant 2 (NCBI: NM_000457.6) was the main *HNF4A* variant in these cell lines (Fig. S2a–c), giving rise to the P1-derived HNF4-alpha-1 protein (UniprotKB: P41235-1). P2-derived transcripts only displayed a minor abundance. In LPS-treated dHepaRG or cytokine-treated HepG2 cells, pre-mRNA of *HNF4A* P1 transcripts levels dropped to 10% after 1 h, followed by a significant reduction in mRNA at later timepoints (Fig. 2d), while P2 transcript levels were not significantly changed (Fig. S3a). Hnf4a mRNA and protein levels were also reduced in murine AML12 cells (Fig. S3b, c) albeit to a lesser extent than in human.

ChIP of RNA Polymerase II (Pol II) revealed significant loss of binding at the promoter region and to a lesser extent at the gene body of *HNF4A* P1 after just 1 h of cytokine treatment (Fig. 2e) in human HepG2 cells. These results underscore that downregulation of *HNF4A* transcription is a major contributor of HNF4A loss during the acute-phase response, and the subsequent reduction in HNF4A levels correspond to a rapid turn-over of the short-lived protein. To determine whether the rapid reduction in Pol II occupancy at the *HNF4A* promoter after 1 h of cytokine treatment is a result of its diversion to pro-inflammatory sites, we performed Pol II ChIPs at AP gene promoters. Neither Pol II recruitment nor mRNA levels of *HP* and *SAA1*/2 were increased after 1 h (Fig S3d, e) indicating that the downregulation of HNF4A precedes the induction of AP gene expression. In addition, the murine AP genes *Saa1* and *Hp* were upregulated by 13- and 5-fold, respectively (Fig. S3e), suggesting a correlation of reduced HNF4A abundance and increased AP gene expression in mouse as well.

### HNF4A binding to acute-phase genes is reduced following inflammatory signals

**Fig. 3:**
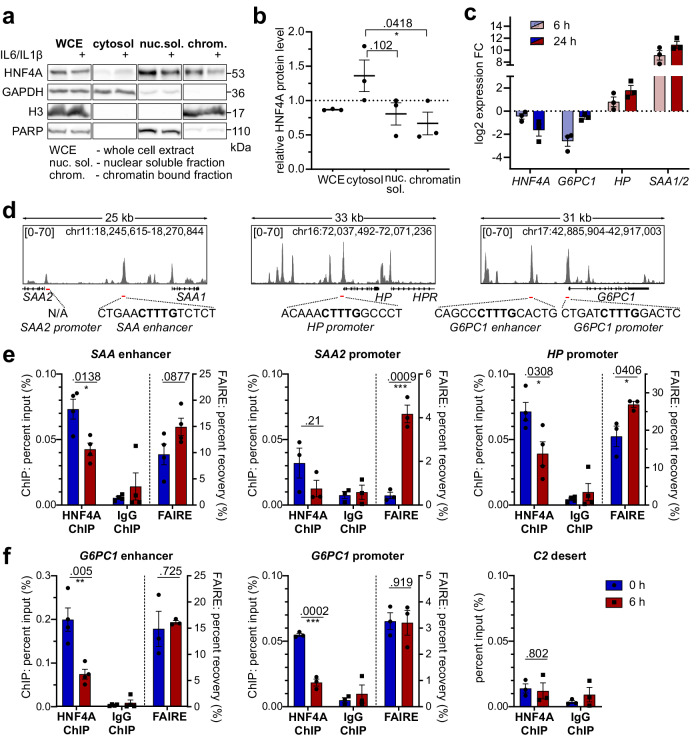
HNF4A chromatin dissociation is associated with de-repression of acute-phase genes. **a** Subcellular fractionation of HepG2 cells treated with IL6/IL1β for 1 h, (**b**) displays densitometric quantification of HNF4A for treated conditions relative to reference protein of each fraction for 3 independent biological replicates: whole cell extract/cytosol-GAPDH, nuclear soluble-PARP, chromatin-H3 (*n* = 3, one-way ANOVA, Post-Hoc: Dunnett’s test). **c** Expression of *HNF4A* (pre-mRNA P1), *G6PC1*, *HP* and *SAA1/2* in HepG2 WT treated for 6 h or 24 h with IL6/IL1β (10 ng/mL), *n* = 3. **d** HNF4A ChIP-Seq peaks (GSE96176)^38^ and HNF4A DNA binding motifs at target regions, amplicons for ChIP- and FAIRE-qPCR are highlighted in red. **e**, **f** HNF4A ChIP and FAIRE in HepG2 WT after 6 h of cytokine treatment (10 ng/mL IL6/IL1β), qPCR was performed at *SAA*, *HP* and *G6PC1* regulatory regions, C2 gene desert served as a negative control (*n* = 3 (SAA2 promoter, G6PC1 promoter, C2 desert and FAIRE), *n* = 4 (SAA enhancer, HP promoter, G6PC1 enhancer), two-tailed unpaired Student’s t-test). Data are expressed as mean ± SEM, significance levels *< 0.05; **< 0.01; ***< 0.001; ****< 0.0001.

Reduced HNF4A abundance has been correlated to loss of liver function^28,30,33^, owing to downregulation of HNF4A target genes that drive homeostatic liver function. Moreover, various signaling pathways that are activated by inflammatory signals are known to phosphorylate HNF4A, thus reducing its DNA binding ability^35–37^. Subcellular fractionation demonstrated a decrease in the chromatin-bound fraction of HNF4A within 1 h of cytokine treatment (Fig. 3a, b). The change in HNF4A chromatin association was associated to a decrease in homeostatic gene expression, e.g. diminished *G6PC1* expression, with a concomitant increase in acute phase gene expression as seen for *HP* and *SAA1/2* (Fig. 3c). In accord with both the rapid impairment of *HNF4A* transcription and its reduced global chromatin association, the HNF4A target gene *G6PC1* (glucose-6-phosphatase catalytic subunit 1) showed diminished expression, following the same kinetics as the rapid decline of *HNF4A* mRNA (Fig. S4a). In addition, stimulation of HepG2 cells with the protein kinase C (PKC) activator phorbol 12-myristate 13-acetate (PMA) and subsequent activation of MAPK pathways resulted in suppression of *HNF4A* transcription comparable to cytokine or LPS stimulation (Fig. S4b, c)

To study the impact of HNF4A activity on acute-phase gene expression we analyzed publicly available ChIP-Sequencing data (GSE96176)^38^ for HNF4A binding in the vicinity of acute-phase genes. Intriguingly, HNF4A binding peaks were found at 27 of the 30 well characterized AP genes^19^ (Fig S7b, Supplementary Data File 1). Subsequently, these peaks were scanned for the HNF4A consensus motif (TGXXCTTTGXXCT) using FIMO^39^ and motifs extracted from the JASPAR database (MA0114.2, MA1494.1, MA0114.4). This analysis revealed a high abundance of HNF4A motifs at AP genes, implying a regulatory role of HNF4A at these genes. *SAA1/2, HP* and *FGG* (fibrinogen gamma chain) displayed the highest relative expression levels in dHepaRG cells (Fig. S5a), and also in HepG2 cells *SAA1/2* and *HP* were strongly responding to treatment. Accordingly, we focused on *SAA1/2* and *HP* for further analysis and used HNF4A-ChIP and FAIRE at regions containing HNF4A binding motifs (Fig. 3d), to test whether the significant upregulation of AP genes could be associated with impaired HNF4A function. The FAIRE assay enriches for nucleosome-free, regulatory DNA elements, reflecting an open chromatin conformation associated with DNase hypersensitivity and histone modifications for active transcription (H3K27ac, H3K4me2/3)^40^.

ChIP-qPCR confirmed HNF4A binding to regulatory regions of *SAA* and *HP* (Fig. 3e-left ChIP) at levels comparable to the *G6PC1* promoter that served as a positive control (Fig. 3f). Upon cytokine treatment, significantly less HNF4A was associated to the *SAA* enhancer, *HP* promoter and *G6PC1* regulatory regions, reiterating the reduced HNF4A DNA binding ability in response to inflammation. Moreover, the increased recovery of chromatin by FAIRE indicates that reduced HNF4A binding correlated with an increasingly accessible chromatin state at both AP gene promoters (Fig. 3e-right FAIRE), permissive for gene transcription. To further test whether HNF4A represses AP genes, *HNF4A* mRNA was knocked down in HepG2 cells that were treated for 6 h with IL6/IL1β (Fig. S5b, c). While the expression of an HNF4A target gene, *G6PC1*, was reduced, the expression of *SAA1/2* was significantly elevated.

These results suggest that HNF4A mediates a basal repression of AP genes which is lifted in response to pro-inflammatory signals, thus allowing robust induction of AP genes.

### Overexpression of HNF4A impedes basal expression of APP genes

**Fig. 4:**
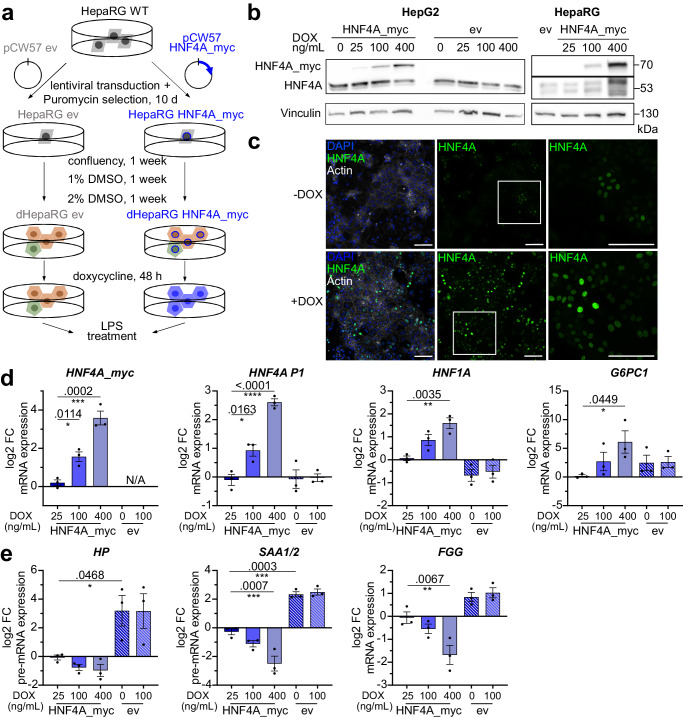
Overexpression of HNF4A in dHepaRG cells represses basal acute-phase gene expression. **a** Generation of stable cell lines for DOX-inducible overexpression of myc-tagged HNF4A. **b** Whole cell protein extracts showing levels of endogenous and myc-tagged HNF4A after 48 h of DOX treatment in HepG2 and HepaRG cell lines. *n* = 3. **c** Immunofluorescence images of HNF4A (green) in HepaRG HNF4A_myc cell line under untreated and doxycycline-treated (400 ng/mL, 48 h) conditions, scale bar = 100 µm. **d** DOX-dependent increase in expression of *HNF4A_myc*, endogenous *HNF4A P1*, *HNF1A* and *G6PC1*. **e** DOX-dependent decrease in acute-phase genes *HP*, *SAA1/2* and *FGG* in dHepaRG HNF4A_myc and ev cells after 48 h of DOX (25; 100; 400 ng/mL) treatment. Data shown in (**d**, **e**) represent as mean ± SEM from *n* = 3 independent experiments, one-way ANOVA post-hoc Holm-Sidak test, significance levels *< 0.05; **< 0.01; ***< 0.001; ****< 0.0001).

To counteract the reduction in HNF4A levels during the APR, HepaRG and HepG2 cell lines harboring a doxycycline-inducible (DOX) transgene of myc-tagged HNF4A (transcript variant 2, NCBI: NM_000457.6) were generated (HNF4A_myc, Fig. 4a, b).

Exogenous HNF4A_myc was localized to the nucleus and dose-dependent upregulation resulted in an auto-regulatory feedback on the endogenous *HNF4A* P1 transcripts (*p* < 0.0001) (Fig. 4c, d). Moreover, the co-regulated TF *HNF1A* (*p* = 0.0035), as well as the target gene *G6PC1* were upregulated in an HNF4A-dependent manner (*p* = 0.0449) (Fig. 4d), confirming the functionality of the overexpressed transgene.

On the other hand, the basal expression of the acute-phase genes *SAA1/2*, *HP* and *FGG* was repressed with increasing DOX concentrations in the HNF4A_myc but not in the empty vector (ev) control cell line (Fig. 4e). *SAA1/2* (*p* = 0.0007) and *FGG* (*p* = 0.0067) were most significantly affected by a HNF4A overexpression at a dose of 400 ng/mL DOX.

In comparison to the dHepaRG ev cell line, overall APP expression was much lower in the dHepaRG HNF4A_myc cell line possibly due to a higher rate of autocrine IL6-signaling in the ev cell line (Fig. S6a). Furthermore, even in the absence of DOX, the dHepaRG HNF4A_myc cell line had an altered growth and differentiation behavior (Fig.S6b, c), since leaky HNF4A_myc expression forced the commitment into the hepatocyte fate and increased the appearance of hepatocyte clusters in dHepaRG_HNF4A_myc cultures (HNF4A positive, Fig. S6b–d)^41^. Conversely, the ev cell line had a higher proportion of cholangiocytes (HNF4A negative, Fig. S6b–d) which are known producers of IL6, thus explaining the large deviation of basal *IL6* and AP gene expression. Due to this bias further analysis was restricted to the dHepaRG_HNF4A_myc cell line comparing DOX-treated or -untreated conditions.

### HNF4A is a negative regulator of acute-phase gene expression

**Fig. 5:**
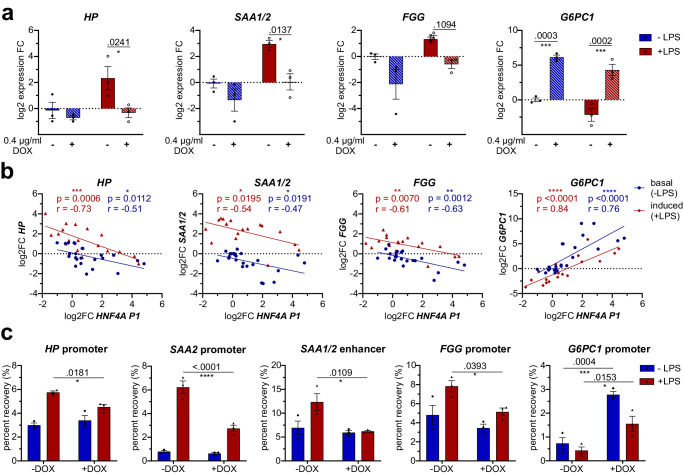
HNF4A retains repressive chromatin state at acute-phase genes. **a** Expression of *HP*, *SAA1/2*, *FGG* and *G6PC1* in dHepaRG HNF4A_myc cells after 6 h LPS treatment (200 ng/mL) and previous DOX-induction (48 h, 400 ng/mL). **b** Pearson correlation and linear regression plot of log2-scaled expression fold changes of target genes against *HNF4A P1* mRNA under basal and LPS-induced conditions in dHepaRG HNF4A_myc cells treated with 0, 25, 100 or 400 ng/mL DOX for 48 h (number of xy pairs basal 24, induced 18, p and r values as indicated). **c** FAIRE-qPCR in dHepaRG HFN4A_myc cell line after 6 h LPS treatment (200 ng/mL) and previous DOX-induction (48 h, 400 ng/mL) at regulatory regions of AP genes. (**a**, **c**) *n* = 3, 2-way ANOVA with post-hoc Sidak’s test). Data are expressed as mean ± SEM, significance levels *< 0.05; **< 0.01; ***< 0.001; ****< 0.0001.

To study HNF4A-dependent inhibition of APP expression in the presence of pro-inflammatory stimuli, a co-treatment of DOX and LPS was performed in the dHepaRG_HNF4A_myc cell line. Indeed, overexpression of HNF4A_myc significantly hindered LPS-induced expression of *SAA1/2* (*p* = 0.0137) and *HP* (*p* = 0.0241) and to a lesser extent also *FGG* (*p* = 0.1094) (Fig. 5a). On the other hand, *G6PC1* expression was boosted (*p* = 0.0003) and HNF4A overexpression restrained LPS-induced reduction of *G6PC1* mRNA (*p* = 0.0002). In addition, *SAA1/2, HP* and *FGG* exhibited a significant negative correlation with *HNF4A* P1 expression levels both under basal and LPS-induced conditions, whereas *G6PC1* showed a strong positive correlation (Fig. 5b).

Consequently, FAIRE assay at the regulatory regions of AP genes was performed to elucidate whether HNF4A-mediated repression alters the chromatin state. LPS treatment for 6 h resulted in a substantial increase in DNA recovery at *SAA1/2*, *HP* and *FGG* regulatory regions. Indeed, the transition to accessible, actively transcribed chromatin was significantly suppressed by HNF4A overexpression at the promoter regions of *HP* (*p* = 0.0181), *SAA2* (*p* < 0.0001) and *FGG* (*p* = 0.0393) (Fig. 5c). Additionally, the enhancer region in between the *SAA1* and *SAA2* genes showed reduced chromatin opening, underlining HNF4A as a repressor of this subset of acute-phase genes. Without LPS addition, chromatin accessibility was unchanged and remained low at APP regulatory regions despite HNF4A overexpression. However, at the *G6PC1* promoter HNF4A-overexpression resulted in higher chromatin accessibility independent of LPS treatment, in line with the increased *G6PC1* expression upon HNF4A gain-of-function (Fig. 5c).

To investigate the mode of HNF4A action at AP gene loci, we performed an extensive motif analysis of publicly available HNF4A ChIP-Seq peaks (human HNF4A ChIP: GSE96176^38^, mouse: GSE90533^17^. This analysis would indicate to what extent the heterogenous group of acute-phase genes is subjected to HNF4A-mediated suppression and whether this is conserved between mouse and human. Among the 30 investigated AP genes 28 showed HNF4A binding peaks, out of which 27 also harbored an HNF4A consensus motif (Fig. S7a). In addition, the HOMER tool was used to identify other TF motifs that were enriched at HNF4A peaks to decipher co-occupying TFs that may either compete or cooperate with HNF4A at these loci (Supplementary Data File 2). Across the entire human and mouse genomes, HNF4A binding sites displayed a strong motif enrichment for Retinoid X Receptor (RXR) homodimers and Peroxisome Proliferator Activated Receptor (PPAR):RXR heterodimers (Fig. S7b), conceivably due to the high conservation of the direct repeat DR1 that is recognized by both RXR and HNF4A dimers^42^. Moreover, motifs of the basic leucine zipper (bZIP) CEBPB (CCAAT enhancer binding protein beta) and related bZIP factors NFIL3 (Nuclear factor, interleukin 3 regulated) and HLF (Hepatic Leukemia Factor) were highly enriched (Fig. S7b). Intriguingly, the motif occurrence for specific factors changed at AP genes compared to their enrichment at the whole genome. In particular, CEBPB, NFIL3, HLF and COUP-TFII (NR2F2, Chicken Ovalbumin Upstream Promoter transcription factor 2) motif abundance increased at HNF4A peaks proximal to AP genes, as seen for HNF1 (hepatic nuclear factor 1) and LRH1 (NR5A2, liver receptor homolog 1) motifs, that have been previously suggested to participate in AP gene regulation (Fig. S7c)^43,44^. CEBPB is a known inducer of AP genes^26^ and may compete with HNF4A binding at target sites. On the other hand, NFIL3 is a known repressor of promoters containing ATF/CREB sites^45^, which are commonly found among the acute-phase genes^46,47^. Moreover, COUP-TFII is a nuclear receptor with repressive functions^48^ that was previously reported to affect HNF4A mode of action^49^. Independent of our motif discovery, the TFs REVERBA (NR1D1, Rev-Erbα) and PROX1 (Prospero homeobox protein 1), which are also associated with transcriptional repression have been suggested to cooperate with HNF4A or even rely on HNF4A-dependent recruitment^17,42,50–52^, thereby determining HNF4A mode of action. Consequently, FIMO motif scanning revealed individual motif occurrence of all factors that were associated with transcriptional repression (PROX1, COUP-TFII, NFIL3 and REVERBA) at HNF4A peaks proximal to AP genes (Fig. S7a, e). Genes, such as *HP* or *FGG*, that were repressed in response to HNF4A overexpression also displayed multiple motifs for REVERBA, NFIL3 or COUP-TF II. Conversely, *CRP* only had a single COUP-TF II motif in the vicinity of the HNF4A motif and did not respond to HNF4A overexpression (Fig. S7d, e).

As the repressive function of these TFs is associated with co-repressor recruitment (e.g. NCoR or HDACs), we analyzed the dependence of HNF4A on co-repressor recruitment in a publicly available ChIP-Seq dataset of HNF4A, HDAC3 and PROX1 in murine liver (GSE90533)^17^. Indeed, a HNF4A-dependent recruitment of both PROX1 and HDAC3 is apparent at murine *Saa1*/*2*, *Hp* and *Fgg* (Fig. S8a) but not at an activated gene such *G6pc* nor at other AP genes as *C3* or *Crp* (Fig. S8b), suggesting a conservation of the HNF4A-mediated effect on a subset of AP genes between species. Consequently, the HDAC-1 and -3 specific inhibitor MS275 was used in combination with LPS and DOX treatments to investigate the role of HDACs in our in vitro model of the hepatic APR (Fig. S8c). In line with reported results^26,44^, MS275 treatment increased both basal and LPS-induced *SAA1/2* expression (Fig. S8d). Intriguingly, the HNF4A-mediated suppression of *SAA1/2* was ameliorated by 1.5-fold by MS275 and the same trend was observed for *HP* – indicating that HDAC-1/-3 are relevant for the HNF4A-mediated repression of those acute-phase genes (Fig. S8d -DOX samples). Conversely, HNF4A overexpression exhibited a repressive effect on both genes also in presence of the HDAC-inhibitor, implying that mechanisms independent of HDAC-1/-3 catalytic activity also contribute to the repression by HNF4A (Fig. S8d +DOX samples).

### HNF4A overexpression retains hepatic functional features while suppressing acute-phase response in a liver organoid model

**Fig. 6:**
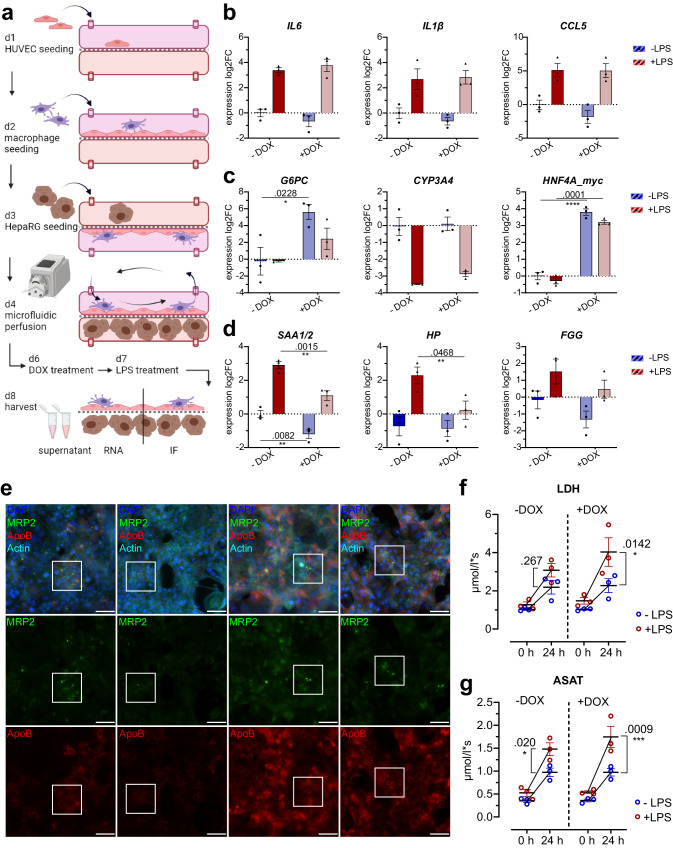
HNF4A overexpression in a microfluidically perfused liver organoid preserves liver functionality during LPS-induced damage. **a** Scheme for seeding, cultivation and treatment with DOX (400 ng/mL, 48 h) and LPS (200 ng/mL, 24 h) of the liver-on-chip organoid system. Created with Biorender.com. **b**–**d** Expression analysis via RT-qPCR of whole liver-on-chip RNA for (**b**) cytokines and chemokines, (**c**) HNF4A_myc and respective targets of liver homeostatic function and (**d**) acute phase genes (*n* = 3, 2-way ANOVA post-hoc Holm-Sidak test). **e** Immunofluorescence of the hepatic layer from the liver-on-chip after the indicated treatments with DOX/LPS, white box indicates highly differentiated hepatocyte clusters, scale = 100 µm. **f**, **g** Measurements of LDH (**f**) and ASAT (**g**) concentrations in hepatic supernatant (*n* = 3, 2-way ANOVA post-hoc Holm-Sidak test). Data are expressed as mean ± SEM, significance levels *< 0.05; **< 0.01; ***< 0.001; ****< 0.0001.

The HepaRG HNF4A_myc cell line was used in a perfused liver organoid model (henceforth termed liver-on-chip) to investigate the effect of HNF4A overexpression in a more physiological, pre-clinical setting. To this end, dHepaRG cells were co-cultivated with primary human umbilical vein endothelial cells (HUVECs) and primary monocyte-derived macrophages in a microfluidically perfused biochip (Fig. 6a), thus robustly enhancing hepatocyte-specific marker gene expression (e.g. hepatocyte polarization marker zonula occludens ZO-1, multidrug resistance-associated protein-2 MRP2, cytochrome P450 monooxygenase CYP3A4) and secretory function (e.g. urea, albumin) compared to a simple 2D culture^53^. Moreover, the liver-on-chip system enables an extensive crosstalk among different cell types resulting in a more complex immune response. An infection via the portal vein was simulated by addition of LPS to the upper cavity, resulting in profuse cytokine secretion by the macrophages and further stimulation of hepatocytes in the lower cavity to mount the acute-phase response.

Expression analysis from bulk liver-on-chip RNA showed comparable expression of *IL6*, *IL1B*, *CCL5* and *CD31* between the DOX-treated and untreated chips (Fig. 6b, Fig. S9a). In the DOX-treated liver-on-chips *HNF4A* was significantly induced (*p* < 0.0001) which was accompanied by increased *G6PC1* levels (*p* = 0.0228, Fig. 6c). Consistent with our observation in 2D cultures, acute-phase gene expression was robustly impaired in the HNF4A-overexpressing liver-on-chip (Fig. 6d). Both *SAA1/2* (*p* = 0.0015) and *HP* (*p* = 0.468) expression were significantly reduced by DOX-treatment, confirming that HNF4A negatively regulates the hepatic acute-phase response in a complex, physiologically relevant environment.

Immunofluorescence detection of the bile acid channel encasing transporter protein MRP2 and the lipid transporter APOB revealed highly differentiated clusters of dHepaRG cells that displayed strong APOB expression and formation of rounded bile acid canaliculi (Fig. 6e, white box). Both APOB and MRP2 signals were diminished, and cell layer integrity was severely disrupted in the LPS-treated liver-on-chip without HNF4A overexpression. Furthermore, inflammation-induced cell damage was detected by increased LDH (lactate dehydrogenase) and ASAT (aspartate aminotransferase) release into the supernatant (Fig. 6f, g), which was detectable also in the upper, endothelial compartment (Fig. S9b, c). DOX treatment substantially increased APOB expression even after LPS treatment (Fig. 6e, S9d), implying that the hepatocytes remained in a differentiated and hence functional state. The internalization of MRP2 and consequent disruption of bile acid transport is a known clinical symptom upon acute hepatitis or sepsis, leading to cholestasis^54^. The MRP2-harboring bile acid channels remained intact in the DOX + LPS co-treated liver-on-chip (Fig. 6e, S9d). This result indicates that HNF4A overexpression might prevent the development of cholestasis resulting from acute inflammation.

Altogether, the sustained *G6PC1*, APOB and MRP2 expression in the DOX + LPS co-treated liver-on-chip indicated that homeostatic liver gene expression was maintained. However, ASAT and LDH concentrations were comparably elevated in both LPS-treated liver-on-chips regardless of HNF4A overexpression (Fig. 6f, g). Inflammatory stress and cell damaging agents which are released by macrophages (ROS, TNFα) are known to throttle the network of LETFs and drive liver failure. Although cell damage or death could not be prevented, HNF4A-overexpressing hepatocytes retained functional integrity.

## Discussion

In this study we report a novel role of the transcription factor HNF4A in the reprogramming of hepatic function during the acute-phase response.

Inflammatory signals suppressed HNF4A function by promptly impeding its DNA binding ability and consequently repressing both transcription of HNF4A itself as well as genes involved in metabolic and vital liver function. Likewise, PMA-treatment led to a rapid and robust repression of HNF4A pre-mRNA (Fig. S4b), rendering PKC and downstream kinases as important mediators of the transcriptional suppression. Phosphorylation of HNF4A is a major contributor to reduced DNA binding ability, and is reportedly induced by various kinases, including GSK3B^55^, AMPK^56^, PKA^36^, PKC^35^ or MAPK^57^. Moreover, stress signals activating ERK, JNK or SRC kinases were shown to diminish either transcription or protein stability of HNF4A^37,58,59^. The complexity of signaling pathways involved underline that regulation of HNF4A transcriptional activity is an important hub in adapting the hepatic transcriptome and metabolic state to environmental cues. Accordingly, the regulation of HNF4A activity seems to be conserved across species. In murine AML12 cells, HNF4A abundance was reduced in response to LPS treatment, enabling increased expression of AP genes (Fig. S3b, c, e). The rapid transcriptional repression could however not be observed in the murine cell line, possibly owing to the low conservation of the human and murine HNF4A promoter sequence.

In this context, our results describe the rapid kinetics of the human HNF4A transcriptional attenuation, which limits HNF4A abundance and activity. The rapid loss of RNA-Pol II from the HNF4A promoter may be due to inactivation of the network of the LETF network controlling HNF4A transcription, including HNF1A, FOXA2, GATA6, HNF6, CEBPA and HNF4A itself^5^. As HNF4A transactivates its own promoter, loss of HNF4A DNA binding reinforces the inhibition of its expression in an autoregulatory manner^6,12^, resulting in a rapid decline of HNF4A levels due to its low half-live. This finding is corroborated by upregulation of endogenous *HNF4A* mRNA upon HNF4A_myc overexpression.

By inducible overexpression of HNF4A in dHepaRG cells, we demonstrated that both the basal and LPS-induced expression of the acute-phase genes *SAA1/2*, *HP* and *FGG* was potently repressed by HNF4A. As inflammatory stimuli limit HNF4A DNA binding ability to boost AP gene induction, HNF4A abundance and activity might serve as a gatekeeper for AP gene expression. How HNF4A exerts its repressive function and to what extent the heterogenous group of AP genes is affected by HNF4A as repressor remains to be clarified. HNF4A binding sites were identified in the vicinity of almost all of the 30 well-described acute-phase genes. However, motifs for other TF, including repressive factors such as COUP-TFII, NFIL3 or REVERBA, varied a lot among the AP genes. The mode of action for HNF4A thus seems to rely on the complex combinatorial interaction with other TFs and co-factors, indicating that HNF4A overexpression might not affect all AP genes in the same way. *CRP*, for instance, requires the HNF4A co-regulated TFs HNF1A and CREBH for its transactivation^43,47^ and was therefore not repressed upon HNF4A overexpression (Fig. S7d). Likewise, PGC1A supports HNF4A transactivation of *SERPINA1* (alpha-1 antitrypsin), where PGC1A retains HNF4A DNA binding and co-activator recruitment also upon cytokine treatment^60,61^.

We demonstrate that HNF4A represses a subset of AP genes by retaining the chromatin at their regulatory regions in an inaccessible state. HNF4A possesses an inherent repressive F-domain at its C-terminus^14,15^, that is reportedly required for transrepression of CLOCK:BMAL-regulated genes and recruitment of co-repressors^16^. Moreover, previous reports showed that HDAC-1 or -3 repress *HP* or *SAA* expression but dissociate from *HP*/*SAA* loci upon pro-inflammatory signals^26,44^. Accordingly, we observed HNF4A occupancy at these genes and a partial reversal of HNF4A-mediated repression by HDAC-1/-3 specific inhibitor MS275. Besides, motif analysis of HNF4A ChIP-Seq peaks proximal to AP genes identified a relative enrichment of TFs that are known to recruit co-repressors and are hence associated with gene repression, such as NFIL3, COUP-TFII or REVERBA. Moreover, Armour *et al* demonstrated in conditional HNF4A knock-out mice an HNF4A-dependent recruitment of PROX1 and HDAC3^17^ which we found to also apply to the subset of AP genes that were repressed by HNF4A (Fig. S8a). However, at other AP genes, such as *C3* or *Crp*, HNF4A did not affect the recruitment of the repressive PROX1/HDAC3 module. This gene-specific recruitment of co-repressors cannot solely be attributed to HNF4A binding sites, but seems to integrate a complex regulatory network of HNF4A, other TF and co-repressors within the chromatin context. Whether the occurrence of repressive factor motifs, like those of NFIL3 or COUP-TFII, at AP genes licenses HNF4A-mediated repression requires further investigation. For instance, deletion of HNF4A’s repressive F-domain would be insightful with regard to co-repressor recruitment. Altogether, HNF4A seems to act as a platform for hierarchical recruitment of co-repressors either directly or indirectly by other LETFs^4^.

Finally, our liver organoid experiments aimed at investigating the suppression of APPs by HNF4A in a pre-clinical setting. Recent studies have underlined the beneficial effects of re-establishing HNF4A expression under chronic pathological conditions^62–64^ which restored the network of LETFs and hence liver function. HNF4A overexpression in the liver-on-chip recapitulated these findings and further affirm HNF4A as a potent repressor of a subset of acute-phase genes. The repression of homeostatic liver functions has been suggested as a general mechanism to reallocate cellular resources to stress response pathways and is strongly correlated to downregulation of LETFs^31,33^. Loss of LETFs function and concomitant transcriptome reprogramming has been revealed in ER stress response^37,47^, damage response to allow regeneration of hepatocytes^32,65^ but also in terms of hepatitis^66^. We show that this concept also applies to the hepatic acute-phase response where inflammation-mediated HNF4A suppression is a prerequisite to induce acute-phase gene expression. Altogether, the dual function of HNF4A as a transactivator and transrepressor renders it as a crucial balancing factor controlling adaptations of the hepatic transcriptome to inflammation.

## Materials and methods

### Cell lines and treatments

HepG2 (DSMZ, #ACC 180) were cultured in RPMI-1640 medium (Sigma-Aldrich) supplemented with 10% FBS (Capricorn), 1000 U penicillin/ 100 µg/mL streptomycin (1% P/S) (Sigma-Aldrich) up to 80–90% confluency and sub-cultured twice or thrice a week at a split ratio of 1:4 up to passage 20. HepaRG cells (Biopredic International, Saint-Grégoire, France) were grown in William’s medium E (Gibco, 22551) supplemented with 10% FBS, 1% P/S, 2 mM L-glutamine (Life Technologies, 25030-024), 5.3 µg/mL insulin (Sigma-Aldrich, I9278-5 ml), and 50 µM hydrocortisone (Sigma-Aldrich, H2270) for 1–2 weeks at confluency. Splitting or seeding of HepaRG was done at a 1:6 ratio once every 1–2 weeks up to passage number 20. HepaRG were differentiated over 2 weeks by addition of 1% (week 1) -2% (week 2) DMSO after growing to 100% confluency for at least one week. AML12 were cultured in DMEM:F12 media (PAN Biotech) with 10% FCS and 1x ITS solution (ITS solution: 10 µg/ml insulin, 5.5 µg/ml transferrin, 5 ng/ml of sodium selenite, 40 ng/ml Dexamethasone) up to 80-90% confluency. They were sub-cultured at a ratio of 1:4 to 1:5 every 2-3 days. Treatment reagents were added to the cell culture medium at the desired concentrations: LPS 100-200 ng/mL (Sigma-Aldrich, N0636), IL6 10 ng/mL (Immunotools, 11340064), IL1β 10 ng/mL (Immunotools, 11340013), Phorbol-12-myristate-13-acetate/PMA 20 nM (Sigma-Aldrich, P8139), MG-132 10 µM (Sigma-Aldrich, C2211), Cycloheximide/CHX 10 µM (Roth, 8682.1), heat-inactivated *E. coli* (XL1 Blue) at final optical density of 0.1, doxycycline DOX 25-500 ng/mL (Sigma-Aldrich, D5207), MS275 1 µM (APExBIO, A8171)

### Transcriptome analysis

Total RNA of each sample was extracted using TRIzol Reagent (Ambion, Life Technologies) and sent for Illumina HiSeq service at Genewiz (now Azenta Life Sciences). Genewiz carried out data processing including quality control (cutadapt v1.9.1) and mapping (Hisat2 2.0.1). The corresponding data were uploaded to the Galaxy web platform for further analysis (usegalaxy.eu^67,68^. Differentially expressed genes were determined with DeSeq2^69^ and filtered for significance (FDR < 0.05) and absolute log_2_ fold change > 1 (see GSE230325). Gene ontology enrichment was performed using GoSeq against the background list of all genes that were detected in the RNA-Seq. GoSeq was used to enrich for biological processes and KEGG pathways^70^ with p values calculated using the Wallenius method and correction for multiple hypothesis testing adjusting the false discovery rate by Benjamini and Hochberg.

Furthermore, transcription factor enrichment was performed with the CheA3 web tool (maayanlab.cloud/chea3/)^34^.

### Motif analysis

Transcription factor enrichment for analysis of over-represented motifs within target sequences was performed with the Galaxy web tool “findMotifsGenome”, which utilizes the HOMER motif analysis software. Analysis was performed on BED files of publicly available HNF4A ChIP-Seq datasets (human HNF4A ChIP: GSE96176, mouse HNF4A ChIP: GSE90533). For analysis of motifs within HNF4A peaks proximal to AP genes, BED files were annotated with the Galaxy web tool “annotatePeaks” which assigns the nearest genomic feature. Peaks proximal to AP genes were selected and also subjected to the “findMotifsGenome” tool. Selected motifs were then scanned for their individual occurrence in the target sequences using FIMO and position weight matrices for each motif provided from the JASPAR database. Data generated can be found in Supplementary Data File 1 and 2.

### Lentiviral transduction

A second-generation plasmid system was used to produce lentiviral particles in HEK293T cells. HEK293T were transiently transfected using the Calcium-Phosphate method with 10 µg psPAX2 (Addgene #12260), 2 µg pMD2.G (Addgene, #12259) and 10 µg of transfer plasmid pCW57-MCS1-2A-MCS2 (Addgene #41393). The HNF4A_myc containing vector was created by subcloning the coding sequence from pcDNA5 FR_HNF4A2 (Addgene, #31100, transcript variant 2, NCBI: NM_000457.6) into the pCW57 backbone. Lentiviral particles were harvested 24 h–48 h post transfection, concentrated using an Amicon-Ultra centrifugal filter (30 kDa cutoff), and used to transduce HepG2 and HepaRG cells. Transduction was supported by addition of 8 µg/mL Polybrene (Sigma-Aldrich, TR-1003-G) and centrifugation at 500 g for 30 min (37 °C). One day post transduction, selection was carried out with 2.5 µg/mL Puromycin (Sigma-Aldrich, P8833) over 10 days. Colonies were singularized, expanded, and resulting cell lines were analyzed for doxycycline-inducible expression of HNF4A_myc.

### RNA interference

HepG2 cells were transiently transfected with 40 nM siRNA (ON-TARGETplus human HNF4A SMARTpool (Dharmacon, #3172), negative control siRNA#1 (Life Technologies, F4611G) using Lipofectamine® 3000 (Invitrogen, L3000-008) according to manufacturer’s instruction. Cells were monitored and harvested 48–72 h post transfection.

### RT-qPCR

RNA was isolated using TRIzol Reagent (Ambion, Life Technologies) and concentration and purity were assessed using a Nanodrop spectral photometer (ND-1000 PeqLab/VWR). CDNA was prepared using DNAseI-digested RNA (Invitrogen, DNaseI Kit) and equal concentrations of Oligo(dT)_18_ and random hexamer primers to amplify complementary mRNA as well as pre-mRNA. RT-qPCR was performed on the StepOne Plus Real-Time PCR System (Applied Biosystems) using PowerUp™ SYBR™ Green Master Mix (Applied Biosystems, A25743) according to manufacturer’s protocol. The primer sequences were designed to span an exon-exon boundary for mRNA and intron-exon boundary for pre-mRNA amplicons. They were previously tested for equal efficiencies and can be found in supplementary Table S3. Expression data was normalized using the ΔΔCt method, calculated with the mean ΔCt for the control conditions.

### Chromatin Immunoprecipitation (ChIP) and Formaldehyde-assisted isolation of regulatory elements (FAIRE)

Chromatin immunoprecipitation was performed as in Bierhoff et al., 2014 and Iyer-Bierhoff et al.,^71,72^ with some modifications. Cells were fixed on their growth plates by addition of 1% formaldehyde to the cell culture medium for 10 min. Fixation was stopped by 0.3 M glycine for 5 min. For ChIP, cell pellets were lysed 30 min in buffer A (100 mM Tris-HCl pH 8.1, 10 mM DTT), and each 5 min in buffer B (10 mM HEPES pH 7.5, 10 mM EDTA, 0.5 mM EGTA, 0.25% (v/v) Triton X-100) and C (10 mM HEPES pH 7.5, 200 mM NaCl, 10 mM EDTA, 0.5 mM EGTA). For sonication, cells were resuspended in buffer D (50 mM Tris-HCl pH 8.0, 10 mM EDTA, 1% (w/v) SDS). For FAIRE, pellets were consecutively lysed in FAIRE buffer 1 (50 mM HEPES pH 7.5, 140 mM NaCl, 1 mM EDTA, 10% (v/v) glycerol, 0.5% (v/v) NP-40, 0.25% (v/v) Triton X-100), FAIRE buffer 2 (10 mM Tris-HCl pH 8.0, 200 mM NaCl, 1 mM EDTA, 0.5 mM EGTA) and FAIRE buffer 3 (10 mM Tris-HCl pH 8.0, 100 mM NaCl, 1 mM EDTA, 0.5 mM EGTA, 0.1% (w/v) sodium deoxycholate, 0.5% (v/v) N-laurylsarcosine), each for 10 min. Sonication was carried out in the Bioruptor Pico (Diagenode) for 8 cycles of 30 s ON/30 sec OFF pulses. Isolation of accessible, nucleosome free DNA regions was performed with phenol-chloroform-isoamylalcohol (Roth, A156.1) as described by ref. ^73^. DNA was de-crosslinked and purified as described above. Percent recovery was quantified by RT-qPCR.

Chromatin used for ChIP was diluted 5x in dilution buffer (16.7 mM Tris-HCl pH 8.0, 167 mM NaCl, 1.2 mM EDTA, 0.01% (w/v) SDS, 1.1% (v/v) Triton-X100) and incubated with antibody-coupled Protein A + G Dynabeads^TM^ (Invitrogen, 10002D, 10004D) overnight on a rotating wheel. Input quantity was comparable between samples and replicates, since the number of cells used for each condition was kept constant. 4 µg of HNF4A (Invitrogen, MAI-199) and 1 µg of RNA-Pol II (Active Motif, 91151) antibody were used. Afterwards, beads were washed consecutively with low salt wash buffer (20 mM Tris-HCl pH 8.0, 150 mM NaCl, 2 mM EDTA, 0.1% (w/v) SDS, 1% (v/v) Triton-X100), 2-times high salt wash buffer (20 mM Tris-HCl pH 8.0, 500 mM NaCl, 2 mM EDTA, 0.1% (w/v) SDS, 1% (v/v) Triton-X100), LiCl wash buffer (10 mM Tris-HCl pH 8.0, 0.25 M LiCl, 1 mM EDTA, 1% (v/v) NP-40, 1% (w/v) sodium deoxycholate) and 2-times TE buffer. DNA was eluted by addition of 100 mM NaHCO_3_, 1% (w/v) SDS and de-crosslinked overnight at 65 °C with 0.3 M NaCl and 10 µg RNAse A (ThermoFisher Scientific, EN0531). DNA was then purified using the Zymoclean^TM^ Gel DNA Recovery Kit (Zymo Research, D4008). RT-qPCR was used to quantify percentage of input of target regions.

### Whole cell protein extracts

Whole cell extracts were prepared using RIPA buffer (150 mM NaCl, 50 mM Tris-HCl (pH 8.0), 1% (v/v) Triton X-100, 0.5% (w/v) sodium deoxycholate, 0.1% (w/v) SDS, 1x proteinase inhibitor cocktail) added as 2-3x volume to the cell pellet. After 20 min incubation on ice, lysates were sonicated for 5 pulses using the Branson sonifier (40% amplitude, 1 sec pulse). Lysates were cleared by centrifugation at full speed and protein content was measured using the PierceTM BCA Protein Assay Kit (ThermoFisher Scientific). SDS-PAGE and Western blot were performed according to standard techniques with antibodies as listed in the Table S4. All original Western blot images are provided in Fig. S10.

### Subcellular fractionation

This protocol was used to separate cell compartments into a cytosolic fraction, a nuclear extract and chromatin-bound or nuclear soluble protein fractions according to a previously described method^74^. In brief, cell pellets were resuspended in 300 µl of cell lysis buffer A (10 mM HEPES (pH 7.9), 5 mM MgCl_2_, 0.25 M sucrose, 0.1% (v/v) NP-40) and incubated for 7 min on ice. Nuclei were then centrifuged at 6000 g for 10 min (4 °C) and the supernatant was transferred into a new reaction tube as cytosolic fraction which was cleared again by centrifugation at full speed for 10 min. The nuclei were resuspended in 125 µl cell lysis buffer B (10 mM HEPES (pH 7.9), 1.5 mM MgCl_2_, 0.1 mM EDTA, 25% (v/v) glycerol). The remaining 85 µl lysate were incubated 15 min on ice after addition of 15 µl 2.5 M KCl to a final concentration of 300 mM. Centrifugation at 9400 g for 15 min led to separation of the nuclear soluble fraction which was transferred to a new reaction tube. The remaining pellet was resuspended in 35 µl buffer B containing 1 M KCl and incubated for 20 min on ice to extract chromatin-bound proteins. Then, 65 µl of lysis buffer B were added to dilute the salt concentration and the chromatin fraction was sonicated for 10 pulses with the Branson sonifier (40% amplitude, 1 s pulse). A final centrifugation at 9400 g for 15 min (4 °C) resulted in the cleared chromatin fraction. Equivalent amounts of each fraction were loaded onto SDS-Gels, i.e., 10% of each fraction and Western Blot was performed according to standard techniques. A list of used antibodies can be found in Table S4.

### Liver organoid model

Liver organoids were assembled as described previously in ref. ^53^ in biochips (BC002) manufactured by Dynamic42 GmbH (Jena, Germany). For assembly of the liver biochip, 300,000 primary human umbilical vein endothelial cells (HUVECs) were seeded in 250 µl Medium 199 into the upper cavity of the sterilized biochip, resembling liver-sinusoidal endothelial cells. HUVECs were isolated from anonymously acquired umbilical cords according to the Declaration of Helsinki, “Ethical principles for Medical Research Involving Human Subjects” (1964) by the Heller lab (Institute of Molecular Cell Biology, University Hospital Jena, Germany)^75^. The study was approved by the Jena University Hospital Ethics Committee (no. 2023-2894) and donors were informed and gave written consent. HUVECs were cultured in supplemented Medium 199 (Lonza) containing 17.5% FBS, 2.5% autologous serum, 1% P/S, 680 µM L-glutamine, 24.8 µg/mL heparin (Sigma-Aldrich, H9399), 0.25% ECGS (Sigma-Aldrich, E2759) and 80 µM L-ascorbic acid (Sigma-Aldrich, A4544). The following day, 100,000 differentiated primary monocyte-derived macrophages, resembling Kupffer cells, were seeded into the same cavity. Primary monocyte-derived macrophages were isolated from leukocyte concentrates obtained from freshly withdrawn peripheral blood of human volunteers (Institute of Transfusion Medicine, University Hospital Jena, Germany) by the Werz lab (Institute of Pharmaceutical Chemistry, Friedrich Schiller University Jena, Germany)^76^. The experimental protocol was approved by the Jena University Hospital Ethics Committee (no. °5050-01/17) and donors were informed and gave written consent. Adherent monocytes were differentiated to macrophages in RPMI 1640 + 10% heat-inactivated FBS supplemented with 1% P/S, 2 mmol/L L-glutamine and 20 ng/mL GM-CSF (Peprotech, Hamburg, Germany) for 6 days. For obtaining HUVECs and primary macrophages, all ethical regulations relevant to human research participants were followed.

Next, 120,000 dHepaRG HNF4A_myc cells were seeded on the opposite side of the biochip and cultured for one day in DMSO-free William’s Medium E. The biochip was attached to tubing and a peristaltic pump (Ismatec Reglo ICC) which allowed perfusion via the vascular chamber with a flow rate of 50 µL/min. The biochip was cultured with daily medium exchange for 3 days (250 µl Medium 199 for upper cavity, 200 µL DMSO-free William’s medium E in the lower cavity) in a humidified cell incubator (5% CO_2_, 37 °C) before treatment was applied.

### Immunofluorescence and image analysis

Cells were fixed with 4% formaldehyde in PBS for 10 min. Blocking for 1 h at RT (3% (w/v) BSA in PBS-0.1% (w/v) Saponin) was followed by primary antibody incubation overnight in the desired concentration (1:200 HNF4A Abcam #ab92378; 1:250 p65 Santa-Cruz #sc-8008; 1:100 MRP2 Cell Signaling Technology, #4446; 1:50 ApoB Santa-Cruz #sc-13538) in incubation solution (0.1% (w/v) Saponin, 0.25% (w/v) BSA in PBS) in a humid chamber at 4 °C. Following day, subjects were washed 3-times in incubation solution and incubated for 1 h at RT with secondary antibodies (1:200, ThermoFisher Scientific #A-11005, A-11008), Hoechst (1:5000, Sigma-Aldrich, B2261-25MG) and Phalloidin-coupled AlexaFluor A488 or A647 (1:100, ThermoFisher Scientific #A12379, #A22287). After 3 washing steps in PBS, subjects were mounted on microscopic slides with Fluoromount-G^TM^ (Invitrogen, 00-4958-02). Imaging was carried out with the inverted fluorescence microscope Eclipse Ti (Nikon) using 20x (Plan Apo 20x Ph2 DM, NA = 0.8 WD = 1000 µm) or 40x (Plan Apo 40x, NA = 0.9 WD = 250 µm) objectives. Images were acquired with the NIS Elements software.

FIJI (v.2.1.0) was used to quantify fluorescence intensities. ApoB mean fluorescence intensity was read from the histogram of the respective (red) channel and normalized to the mean FI of the DAPI (blue) channel. MRP2 positive foci were determined by thresholding the 8-bit image of the respective channel (green) equally among all analyzed images and using the „Analyze Particles“ command to determine the total area. This was normalized to the total are of nuclei, as described above. For each biological replicate, at least 3 image sections were analyzed.

### ASAT and LDH measurements

The respective parameters were measured in cell culture supernatants using the Cobas 8000 Modular analyzer (Roche Diagnostics International AG, Rotkreuz, Switzerland) according to the manufacturer’s protocol.

### Statistics and reproducibility

Analysis was performed on at least three biological replicates, i.e. three independent experiments performed on three independent days, calculating mean with SEM. Graph-Pad Prism v7.0.5 was used to perform statistical tests as indicated for each result. In general, for comparing one condition among groups a one-way ANOVA was performed, for two conditions among groups a two-way ANOVA with subsequent Holm-Sidak’s multiple comparison testing. Student’s t-test was used to compare two means if multiple comparisons were not required. All *p*-values < 0.05 were considered significant.

### Reporting summary

Further information on research design is available in the Nature Portfolio Reporting Summary linked to this article.

### Supplementary information

Supplementary Material

Description of Additional Supplementary Files

Supplementary Data File 1

Supplementary Data File 2

Supplementary Data File 3

Reporting Summary

## Data Availability

The datasets analyzed in this study were obtained from the Gene Expression Omnibus collection GSE96176 (human HNF4A ChIP-Seq on liver)^38^ and GSE90533 (mouse ChIP-Seq for HNF4A GSM2466339, PROX1 GSM2466337, HDAC3 GSM2466335)^17^. The datasets produced in this study are available in the following databases: RNA-Seq data: Gene Expression Omnibus GSE230325. The source data behind the graphs can be found in Supplementary Data 3. Uncropped and unedited western blot images can be found in Fig.S10.
