# Supplementary material for: Downregulation of HNF4A enables transcriptomic reprogramming during the hepatic acute-phase response
Source: Commun Biol. 2024 May 16;7:589. doi: 10.1038/s42003-024-06288-1 (PMC11099168; doi:10.1038/s42003-024-06288-1)
Supplement: Supplementary file 1 — Supplementary Material [file 42003_2024_6288_MOESM1_ESM.pdf]

Supplementary material

Downregulation of HNF4A enables transcriptomic reprogramming during the hepatic acute-phase response

Charlotte Ehle[1], Aishwarya Iyer-Bierhoff[1], Yunchen Wu[1, 2], Shaojun Xing[2], Michael Kiehntopf[3], Alexander S Mosig[4], Maren Godmann[1], Thorsten Heinzel[1]

[1] Institute of Biochemistry and Biophysics, Center for Molecular Biomedicine, Friedrich Schiller University Jena, 07745 Jena, Germany

[2] Marshall Laboratory of Biomedical Engineering, Department of Pathogen Biology, Shenzhen University Medical School, Shenzhen University, Shenzhen, Guangdong, China. 518060

[3] Department of Clinical Chemistry and Laboratory Diagnostics, Jena University Hospital, 07747 Jena, Germany

[4] Institute of Biochemistry II, Center for Sepsis Control and Care, Jena University Hospital, 07747 Jena, Germany

Table of contents

Table S1 2

Table S2 3

Table S3 4

Table S4 5

Fig. S1 9

Fig. S2 10

Fig. S3 11

Fig. S4 12

Fig. S5 13

Fig. S6 14

Fig. S7 15

Fig. S8 16

Fig. S9 17

Fig. S10 18

Table S1: Expression data of acute-phase genes from the RNA-Seq data set at 0 vs 6h or 0 vs 24 h, bold are significantly upregulated genes (log2 FC >1, FDR <0.05)

Function	Gene symbol	6 h			24 h		
		**log2 FC**	**p value**	**p adj (FDR)**	**log2 FC**	**p value**	**p adj (FDR)**
complement, opsonins, soluble PRRs	C3	-0.126	4.05E-01	5.88E-01	0.705	2.89E-06	6.16E-05
	C4A	-0.228	1.09E-01	2.32E-01	-0.091	5.23E-01	7.57E-01
	C9	N/A	N/A	N/A	N/A	N/A	N/A
	C4BPA	-0.110	5.29E-01	6.96E-01	0.724	2.58E-05	4.25E-04
	SERPING1	-0.252	5.32E-02	1.35E-01	-0.141	2.81E-01	5.48E-01
	**CRP**	**4.322**	**5.38E-69**	**2.67E-66**	**5.411**	**4.76E-108**	**8.71E-105**
	**SAA2**	**1.713**	**8.99E-35**	**1.60E-32**	**3.213**	**4.78E-118**	**1.12E-114**
	SAA1	N/A	N/A	N/A	**2.210**	**1.24E-65**	**8.88E-63**
	APCS	-0.851	2.26E-02	6.88E-02	0.524	1.60E-01	3.94E-01
	**PTX3**	**4.815**	**1.54E-99**	**1.53E-96**	**5.166**	**1.11E-114**	**2.28E-111**
	FN1	0.340	5.38E-03	2.13E-02	0.761	4.52E-10	2.11E-08
coagulation, clotting cascade	FGA	-0.394	1.76E-02	5.63E-02	0.919	3.01E-08	1.00E-06
	**FGB**	-0.024	8.81E-01	9.37E-01	**1.331**	**2.64E-16**	**2.58E-14**
	**FGG**	-0.133	3.96E-01	5.80E-01	**1.258**	**1.31E-15**	**1.18E-13**
	F11	-0.677	3.95E-02	1.06E-01	-0.096	7.64E-01	8.98E-01
	PLAU	-0.009	9.84E-01	9.92E-01	N/A	N/A	N/A
	**PLAT**	**4.681**	**2.93E-28**	**3.54E-26**	-0.009	9.83E-01	9.94E-01
	VTN	0.086	5.18E-01	6.87E-01	-0.722	6.80E-08	2.15E-06
	PLG	-1.086	3.57E-03	1.52E-02	-0.974	8.95E-03	5.27E-02
	**SERPINE1**	**1.898**	**5.17E-55**	**1.71E-52**	-0.102	4.02E-01	6.67E-01
protease inhibitors	SERPINA1	0.661	8.09E-08	1.24E-06	0.840	9.24E-12	5.57E-10
	**SERPINA3**	**2.544**	**1.28E-29**	**1.70E-27**	**2.837**	**7.84E-37**	**2.69E-34**
	A2M	0.660	3.28E-02	9.25E-02	-0.116	7.07E-01	8.69E-01
heme, metal binders, transporters	**HP**	-0.238	5.17E-01	6.86E-01	**1.257**	**6.30E-04**	**6.46E-03**
	HPX	-0.124	3.27E-01	5.11E-01	-0.386	2.34E-03	1.86E-02
	HAMP	-1.079	1.43E-02	4.77E-02	0.342	4.37E-01	6.95E-01
	**CP**	**1.819**	**5.24E-25**	**5.10E-23**	**3.684**	**5.84E-98**	**8.74E-95**
	**ORM1**	0.160	5.94E-01	7.46E-01	**1.494**	**6.67E-07**	**1.70E-05**
	**ORM2**	0.225	4.73E-01	6.50E-01	**1.564**	**5.93E-07**	**1.52E-05**
signalling, others	CD14	0.604	5.58E-04	3.14E-03	0.486	5.48E-03	3.59E-02
	**LBP**	**1.454**	**5.92E-20**	**4.09E-18**	**2.886**	**9.84E-74**	**8.10E-71**
	**IL1RN**	**2.166**	**1.41E-06**	**1.64E-05**	**1.317**	**3.51E-03**	**2.55E-02**
	SERPINA8	-0.362	3.00E-03	1.31E-02	-0.854	2.51E-12	1.66E-10

Table S2: Overview of HNF4A transcript variants and respective qPCR primers. Main variants of P1/P2 isoforms in bold.

primer name	**proteinatlas** splice variant	transcript variant/NCBI NM	uniprot ID	isoform	primer sequences	exons
HNF4A P1 (1-3)	204	1/ NM_178849.3	P41235-2	P1	fwd: GATGGGCAATGACACGTCC<br>rev: GGAGTACATGTGGTTCTTCCG	1A, 2
	201	**2/ NM_000457.6**	P41235-1	P1		
	205	3/ NM_178850.2	P41235-3	P1		
HNF4A 201	204	1/ NM_178849.3	P41235-2	P1	fwd: CCAACCCAACCTCATCCTCC<br>rev: CAAGTTCCTGTTGCAGTCGC	exon 10 start (shared short P1/P2)
	201	**2/ NM_000457.6**	P41235-1	P1		
	202	4/ NM_001258355.2	P41235-2	P1		
	202	**5/ NM_175914**	P41235-5	P2		
	-	7/ NM_001258355.2	-	P2		
	206	8/ NM_001287182.2	P41235-6	P2		
	209	9/ NM_001287183.2	-	P2		
HNF4A 204	204	1/ NM_178849.3	P41235-2	P1	fwd: CACTGGGCTTCTCTCCAAGG<br>rev: CACAACCCCGAGTGAATGGA	exon 10 end (shared long P1/P2)
	202	4/ NM_001258355.2	P41235-2	P2		
	-	7/ NM_001258355.2	-	P2		
	206	8/ NM_001287182.2	P41235-6	P2		
HNF4A P2 (4-6)	202	4/ NM_001258355.2	P41235-5	P2	fwd: GAGAGTTCTTACGACACGTCC<br>rev: GAGGCACCGTAGTGTTTGC	1D, 2
	202	**5/ NM_175914**	P41235-5	P2		
	208	6/ NM_001030004.3	P41235-7	P2		
HNF4A P2 (8-10)	206	8/ NM_001287182.2	P41235-6	P2	fwd: GGAGAGTTCTTACGGTCTGC<br>rev: TGCACAACCTCTTCATCCT	1D, 1C
	209	9/ NM_001287183.2	-	P2		
	208	10/ NM_001287184.2	P41235-7	P2		

Table S3: RT-qPCR and ChIP/FAIRE-qPCR primers

target	oligo name	sequence (5'-3')
RPL3 mRNA	hRPL3_for	GATACAAGGCTGGCATGACTC
	hRPL3_rev	GTGGTGTCTCTACAATGGTCACA
HNF4A P1 (1-3) mRNA	hHNF4A P1 fwd	GATGGGCAATGACACGTCC
	hHNF4A P1 rev	GGAGTACATGTGGTTCTTCCG
HNF4A P2 (4-6) mRNA	hHNF4A P2 (4-6) fwd	GAGAGTTCTTACGACACGTCC
	hHNF4A P2 (4-6) rev	GAGGCACCGTAGTGTTTGC
HNF4A (201) mRNA	hHNF4A 201 fwd	CCAACCCAACCTCATCCTCC
	hHNF4A 201 rev	CAAGTTCCTGTTGCAGTCGC
HNF4A (204) mRNA	hHNF4A 204 fwd	CACTGGGCTTCTCTCCAAGG
	hHNF4A 204 rev	CACAACCCCGAGTGAATGGA
HNF4A P2 (8-10) mRNA	hHNF4A P2 fwd	GGAGAGTTCTTACGGTCTGC
	hHNF4A P2 rev	TGCACAACCTCTTCATCCT
HNF4A P1 pre-mRNA	HNF4A P1 pre fwd	GCCTCACTCCCTTCTCTCCT
	hHNF4A P1 rev	GGAGTACATGTGGTTCTTCCG
HNF4A_myc mRNA	HNF4A-myc qPCR	ATAGCGCGGCCGCCACTGTG
	pHNF4A rev col2	TCGCATGGTACCAGGCCTTG
HP mRNA	qPCR_HP (for)	AATGTGAAGCAGTATGTGGG
	qPCR_HP (rev)	CTGTGGTGAGATTATGGTGG
SAA 1/2 mRNA	qPCR_SAA (for)	ACAGATCAGCACCATGAAGC
	qPCR_SAA (rev)	TGTCTGAGCCGATGTAATTGG
CRP mRNA	qPCR_CRP (for)	TCGTGGAGTTCTGGGTAGATGG
	qPCR CRP (rev)	TTCCCACCGAAGGAATCCTGCT
IL6 mRNA	IL-6 (fwd)	GGTACATCCTCGACGGCATCT
	IL-6 (rev)	GTGCCTCTTTGCTGCTTTCAC
HNF1A mRNA	hHNF1a fwd	AGACGCTAGTGGAGGAGTGCAA
	hHNF1a rev	GGCAAACCAGTTGTAGACACGC
G6PC mRNA	hG6PC_C_For	GCTGTGATTGGAGACTGGCTCA
	hG6PC_C_Rev	GTCCAGTCTCACAGGTTACAGG
FGG mRNA	qPCR_FGG (for)	CACTGGGAAAGATTGTCAAGAC
	qPCR_FGG (rev)	ATCTACACTGCCATCAAGTCTC
Rpl3 mRNA	mRpl3 fwd	CAGCGATGAGTGTAAAAGGCGC
	mRpl3 rev	GCTGCTTCTTGCCTGTGTCATC
Saa1 mRNA	mSaa1 fwd	GGAGTCTGGGCTGCTGAGAAAA
	mSaa1 rev	TGTCTGTTGGCTTCCTGGTCAG
Hp mRNA	mHp fwd	ACGGCTATGTGGAGCACTTGGT
	mHp rev	GTTTCTCTCCAGCGACTGTGTTC
Hnf4a mRNA	mHnf4a fwd	TTCATCAAGCTCTTCGGCAT
	mHnf4a rev	GTTCTTGCATCAGGTGAGGG
HNF4A P1 promoter	HNF4A_P1 prom fwd	CTGAACATCGGTGAGTTAGG
	HNF4A_P1 prom rev	GGATTTGGCTGTTTGTTGGT

Table S3 (continued): RT-qPCR and ChIP/FAIRE-qPCR primers

HNF4A gene body	HNF4A P1 pre fwd	GCCTCACTCCCTTCTCTCCT
	hHNF4A P1 rev	GGAGTACATGTGGTTCTTCCG
C2 gene desert	hGenedes_C2 fwd	CATCCCTGGACTGATTGTCA
	hGenedes_C2 rev	GGTTGGCCAGGTACATGTTT
RPL3 promoter	RPL3_prom fwd 1	TCAAATCCCGCCGGTAGAG
	RPL3_prom rev 1	CGAAAGCTAGGAGCGGAAGA
HP promoter	ChIP_HP-prom_fwd	AATACTTTGGCAGGTTTGTGG
	ChIP_HP-prom_rev 3	GCTCTTGCTTCACACTTGATTT
SAA2 promoter	ChIP_SAA2-prom_fwd	CTCTTGTTCCCATAGGTTACAC
	ChIP_SAA2-prom_rev	TTTATAGTGAGCCTTGCTGGT
SAA enhancer	SAA1 HNF4-motif fwd 1	GGAACTCAGTGTCACAACCT
	SAA1 HNF4-motif rev 1	CAGCAGAAACCTGAACTTTGTC
G6PC promoter	hG6PC_prom-fwd1	CATTGGCCCTGCTGAGTACA
	hG6PC_prom-rev1	AACCCAGCCCTGATCTTTGG
G6PC enhancer	hG6PC_distenh1-fwd1	CCTTTGCACTGGCGAACAAT
	hG6PC_distenh1-rev1	TGGTGGACATGGGAAACTGG

Table S4: List of reagents and tools

Reagent/resource	reference or source	Indentifier or cat. #
**experimental models**		
HepaRG	Biopredic International (Gripon et al. 2002)	-
HepG2	DSMZ	#ACC 180
HUVEC	Regine Heller, UKJ Jena (Spengler et al. 2020 PMID: 32168879)	-
primary monocyte-derived macrophages	Oliver Werz, FSU Jena (Pace et al. 2017 PMID: 28737505)	-
AML12	Ignacio Rubio, university hospital Jena	ATCC #CRL-2254
**recombinant DNA**		
psPAX2	Addgene	#12260
pMD2.G	Addgene	#12259
pcW57.1 MCS1-2A-MCS2	Addgene	#41393
pcDNA5 FR_HNF4A2	Addgene	#31100
pcDNA3 HNF4A2_myc	Charlotte Ehle	-
pcW57_HNF4A2_myc	Charlotte Ehle	-

Table S4 (continued):

Antibodies	reference or source	Indentifier or cat. #	concentration
anti-mouse HRP conjugate secondary antibody	Jackson Immunoresearch	115-035-062	1:5000 (WB)
anti-rabbit HRP conjugate secondary antibody	Jackson Immunoresearch	115-035-144	1:5000 (WB)
anti-HNF4A (for ChIP)	Invitrogen	MAI-199	4 µg (ChIP)
anti-HNF4A (for WB)	Abcam	ab181604	1:2500 (WB)
anti-Vinculin	Bio-Rad	MCA465GA	1:5000 (WB)
anti-Histone H3	Abcam	ab1791	1:10,000 (WB)
anti-Actin	Sigma-Aldrich	A2066	1:50,000 (WB)
anti-phospho-ERK (Thr202/Tyr204)	Cell Signaling Technology	9101	1:2000 (WB)
anti-ERK	Santa-Cruz	sc-93	1:1000 (WB)
anti-P-PKC Motif antibody, MultiMab™ [(R/K)XpSX(R/K)]	Cell Signaling Technology	6967P	1:1000 (WB)
anti-GAPDH	Cell Signaling Technology	85925	1:5000 (WB)
anti-PARP	Cell Signaling Technology	9542	1:1000 (WB)
anti-ApoB	Santa Cruz	sc-13538	1:50 (IF)
anti-MRP2/ABCC2	Cell Signaling Technology	4446	1:100 (IF)
anti-RNA Pol II	Active Motif	91151	1 µg (ChIP)
rabbit IgG	Cell Signaling Technology	2729	1 µg (ChIP)
mouse IgG	Merck	12-371	1 µg (ChIP)
Goat anti-Mouse IgG (H+L) Cross-Adsorbed Secondary Antibody, Alexa Fluor™ 594	Thermo Fisher Scientific	A-11005	1:200 (IF)
Goat anti-Rabbit IgG (H+L) Cross-Adsorbed Secondary Antibody, Alexa Fluor™ 488	Thermo Fisher Scientific	A-11008	1:200 (IF)

**Oligonucleotides and sequence-based reagents**		
qPCR primers		see Table S1
ON-TARGETplus human HNF4A SMARTpool	Dharmacon	#3172
**chemicals, enzymes and other reagents**		
RPMI-1640 Medium	Sigma-Aldrich	R8758
Dulbecco's Modified Eagle Medium (DMEM + GlutaMAX™)	Gibco	61965-026
Opti-MEM + GlutaMAX™	Gibco	51985-026
William's Medium E (WE)	Gibco	22551
Medium 199 (M199)	Lonza	CE12-117F
DMEM/F12	PAN Biotech	P04-41250

Table S4 (continued):

**chemicals, enzymes and other reagents**		
Dulbecco's Phosphate Buffered Saline (PBS)	Sigma-Aldrich	D8537
Trypsin-EDTA Solution	Sigma-Aldrich	T3924
Fetal Bovine Serum (FBS)	Capricorn	FBS-11A
Penicillin/Streptomycin (10,000 U penicillin/ml, 10 mg streptomycin/ml)	Sigma-Aldrich	P4333
L-Glutamine (200 mM)	Life Technologies	25030-024
Insulin human, BioXtra	Sigma-Aldrich	I9278-5ml
Hydrocortisone 21-hemisuccinate sodium salt	Sigma-Aldrich	H2270
heparin sodium salt (≥180 USP U/mg)	Sigma-Aldrich	H9399
endothelial cell growth supplement (ECGS)	Sigma-Aldrich	E2759
L-ascorbic acid	Sigma-Aldrich	A4544
ITS solution	Sigma-Aldrich	I3146
IL1β	Immunotools	11340013
IL6	Immunotools	11340064
LPS (Escherichia coli O111:B4)	Sigma-Aldrich	L4391
MG-132	Sigma-Aldrich	C2211
MS275	APExBIO	A8171
Phorbol-12-myristate-13-acetate (PMA)	Sigma-Aldrich	P8139
Polybrene	Sigma-Aldrich	TR-1003-G
Puromycin	Sigma-Aldrich	P8833
Cycloheximide (CHX)	Roth	8682.1
Doxycycline (DOX)	Sigma-Aldrich	D5207
Dynabeads™	Invitrogen	10002D, 10004D
Hoechst	Sigma-Aldrich	B2261
Fluoromount-G™	Invitrogen	00-4958-02
Lipofectamine® 3000	Invitrogen	L3000-008
**Software**		
cutadapt	https://cutadapt.readthedocs.io/en/stable/	v1.9.1
Hisat2	Sirén et al. 2014	2.0.1
Galaxy.eu using<br>DESeq2<br>GoSeq	Galaxy Community. 2022 PMID: 35446428;<br>Love et al. 2014 PMID: 25516281;<br>Young et al. 2010 PMID: 20132535	
CheA3	maayanlab.cloud/chea3/<br>Keenan et al. 2019<br>PMID: 31114921	

Table S4 (continued):

**Software**		
Cistrome DB	http://cistrome.org/db/#/<br>Liu et al. 2011<br>PMID: 21859476	
FIMO	https://meme-suite.org/meme/tools/fimo<br>Grant et al. 2011<br>PMID: 21330290	V5.5.3
FIJI	Schindelin et al. 2019<br>PMID: 22743772	2.1.0
GraphPad Prism	Dotmatics	8.0.2
StepOne Software	Applied Biosystems	v2.3
Fusion	Vilber Lourmat	
**Equipment**		
Bioruptor Pico	Diagenode	
Biochip BC-002	Dynamic-42	
Microscope, Nikon Eclipse Ti	Nikon	
qRT-PCR Machine, StepOne Plus	Applied Biosystems (ThermoFisher Scientific)	
peristaltic pump, ISMATEC Reglo ICC	ISMATEC	
Cobas 8000 Modular analyzer	Roche Diagnostics International AG	

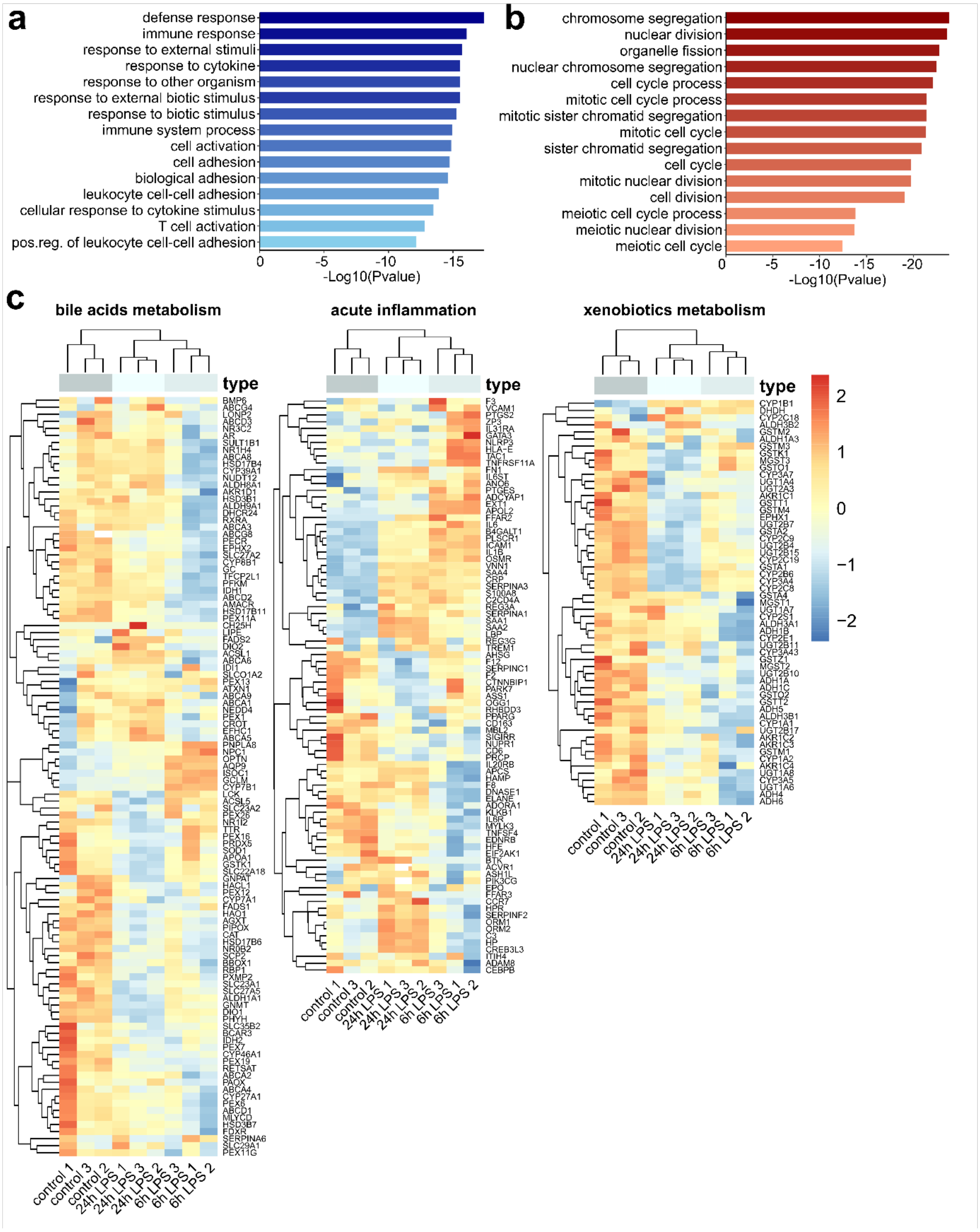

Fig. S1: Dynamic changes in the hepatic transcriptome.
Top 15 gene ontologies (KEGG, biological process) of downpregulated (a) or upregulated (b) gene set for the comparison 6h vs 24h. (c) Heatmaps for selected MSigDB hallmark gene sets “bile acids metabolism”, “xenobitoics” metabolism and the GO biological process “acute inflammatory response” (GO:0002526).

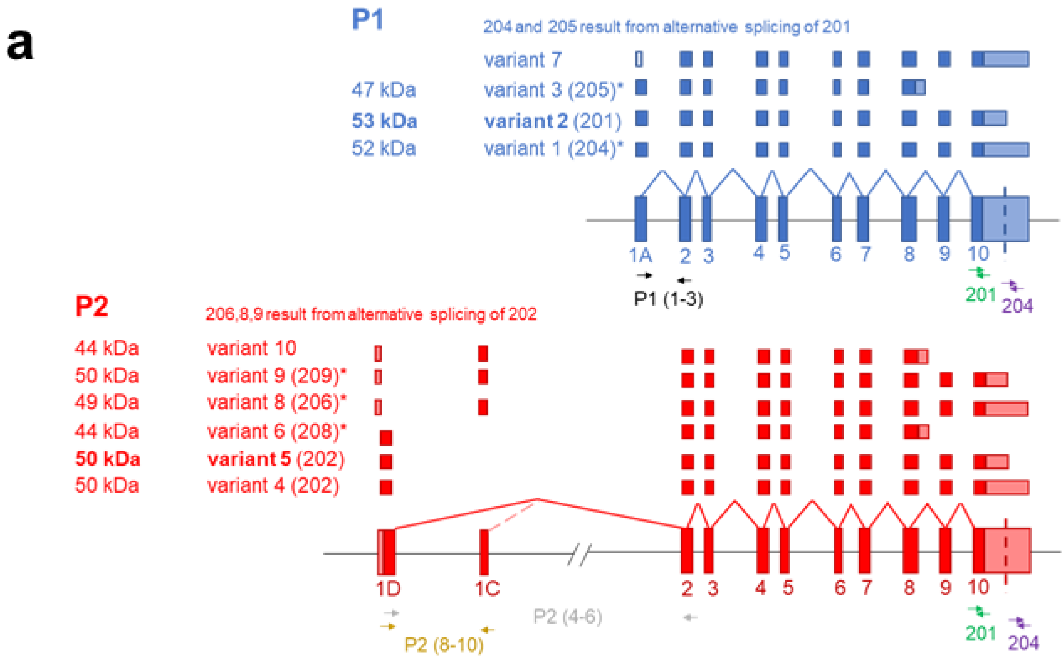

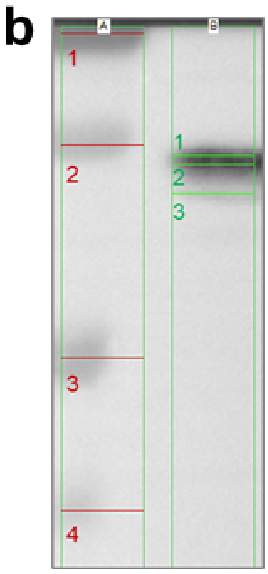

band #	(A) protein ladder in kDa	(B) HNF4A in kDa	suggested isoform
1	70	53,721	P1, transcript variant 2
2	55	52,766	P1, transcript variant 1
3	37	49,858	P2, transcript variant 4/5
4	25		

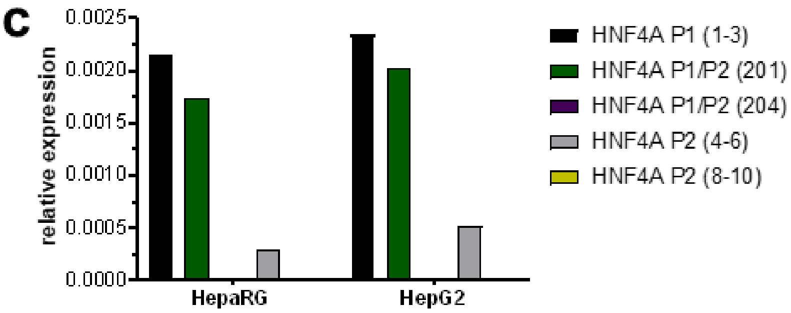

Fig. S2: *HNF4A2* is the main HNF4A transcript variant in HepG2 and HepaRG cells. (a) overview of P1 and P2 derived HNF4A-transcripts (according to NCBI and human proteinatlas) with indicated sizes of gene products and respective exon usage. Primer sequences were designed to specifically differ between P1/ P2 or long/short isoforms (see Table 1, p.8). (b) Estimation of HNF4A protein band sizes in Western blot with Vilber Fusion software. (c) Relative expression of HNF4A transcripts in HepaRG and HepG2 WT cell lines, n=1

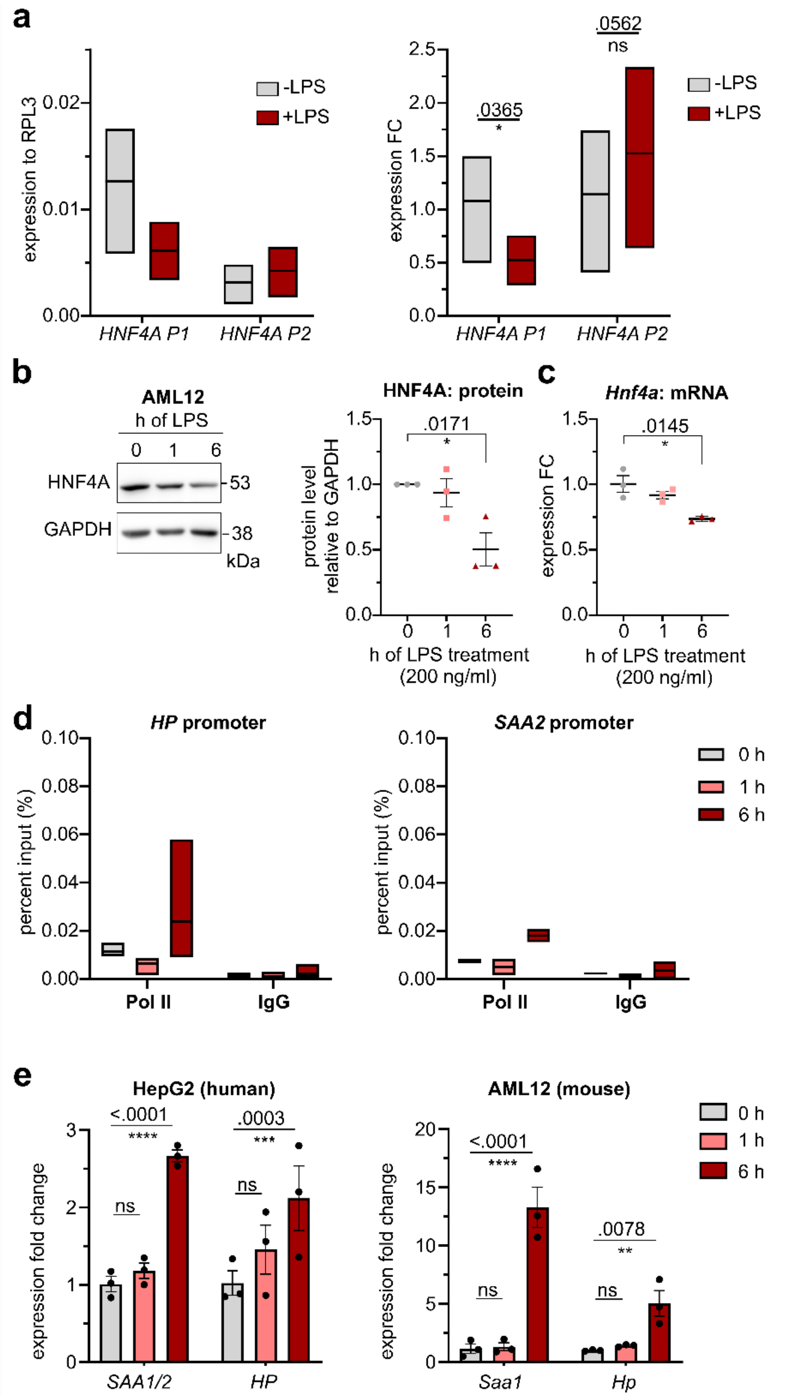

Fig. S3: HNF4A isoform expression, murine Hnf4a expression and AP gene induction after pro-inflammatory stimuli.

(a) Expression levels of HNF4A P1 and P2 isoforms relative to RPL3 and fold change after 6 h LPS (200 ng/ml) in HepaRG WT (n=3, data displayed from min-max, line at mean, 2-Way ANOVA with Holm Sidak's multiple comparison testing). (b) Western blot and densitometric quantification of HNF4A and (c) Hnf4a mRNA expression of AML12 cells treated for 0; 1; 6 h with LPS (200 ng/ml). (b, c) n=3, data displayed as mean ± SEM, Student's T-test comparing 0 and 6 h. (d) ChIP-qPCR of RNA PolII at promoter regions of *HP* and *SAA2*, after 1 and 6 h cytokine (10 ng/ml IL6/IL1β) treatment in HepG2 WT (n = 4 (HP), n = 2 (SAA2), data displayed from min-max, line at mean (e) RT-qPCR of AP genes after 0; 1; 6 h of 100 ng/ml LPS treatment of HepaRG WT (left) or 200 ng/ml treatment of AML12 (right) (n = 3, data displayed as mean±SEM, 2-way ANOVA with Dunnett's correction for multiple comparisons).

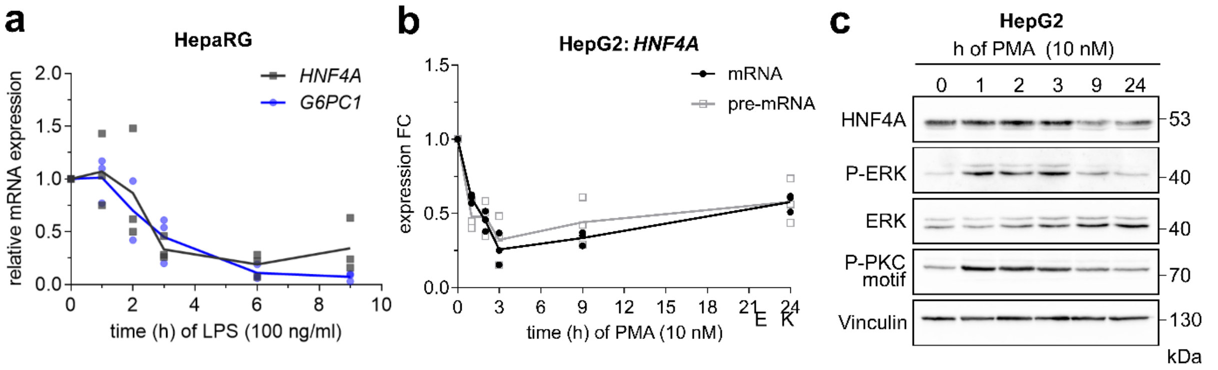

Fig. S4: HNF4A mRNA decline after PKC activation.

(a) Time course of *G6PC1* and *HNF4A* P1 mRNA expression after LPS treatment in dHepaRG WT cells. (b) Time course of *HNF4A* P1 mRNA and pre-mRNA after PMA treatment in HepG2 WT cells. (c) Western blot of HNF4A after PMA treatment and subsequent activation of PKC and ERK kinases. n = 3 (a, b) line at mean.

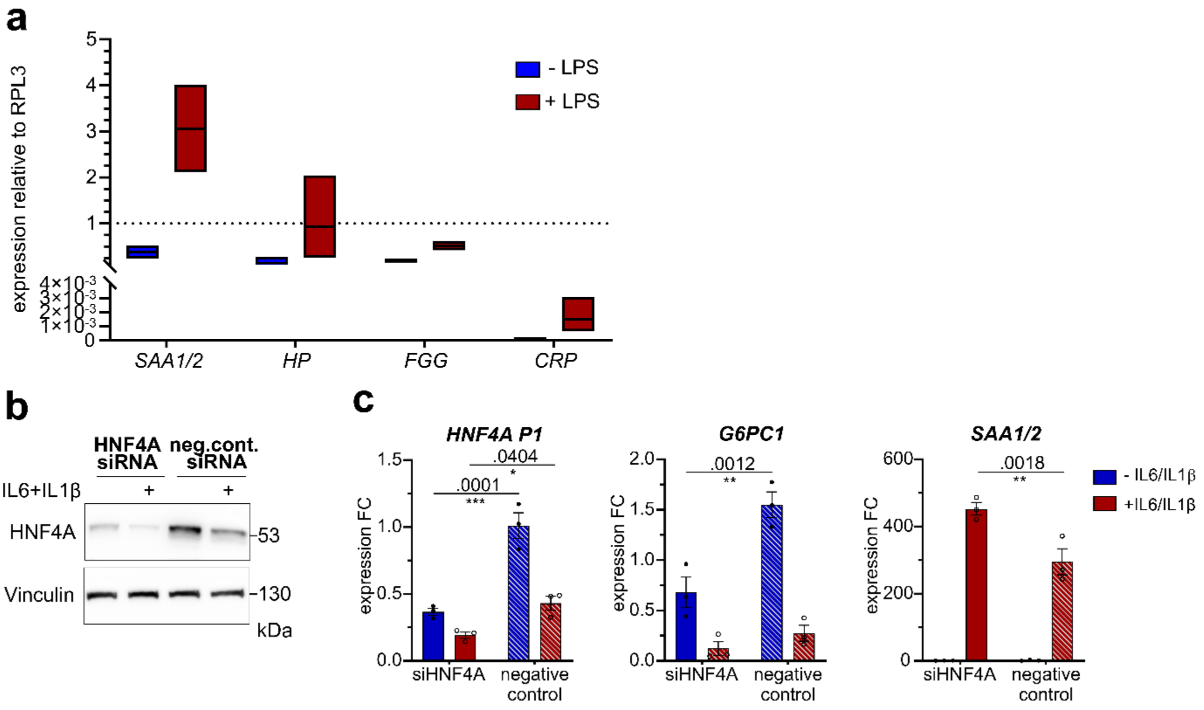

Fig. S5: Relative Expression of AP genes and effects of HNF4A knockdown.
(a) Relative expression of *SAA1/2*, *HP*, *FGG* and *CRP* as compared to housekeeping gene RPL3 (dashed line), n=3, data represented from min to max, line at mean. (b, c) HNF4A knockdown in HepG2 cells with 6 h cytokine treatment. (b) Whole cell protein extracts showing levels of HNF4A after knockdown and cytokine treatment, (c) qRT-PCR results for HNF4A P1, G6PC1 and SAA1/2 expression (n= 3, Data are expressed as mean±SEM 2-way ANOVA, post-hoc Holm-Sidak test).

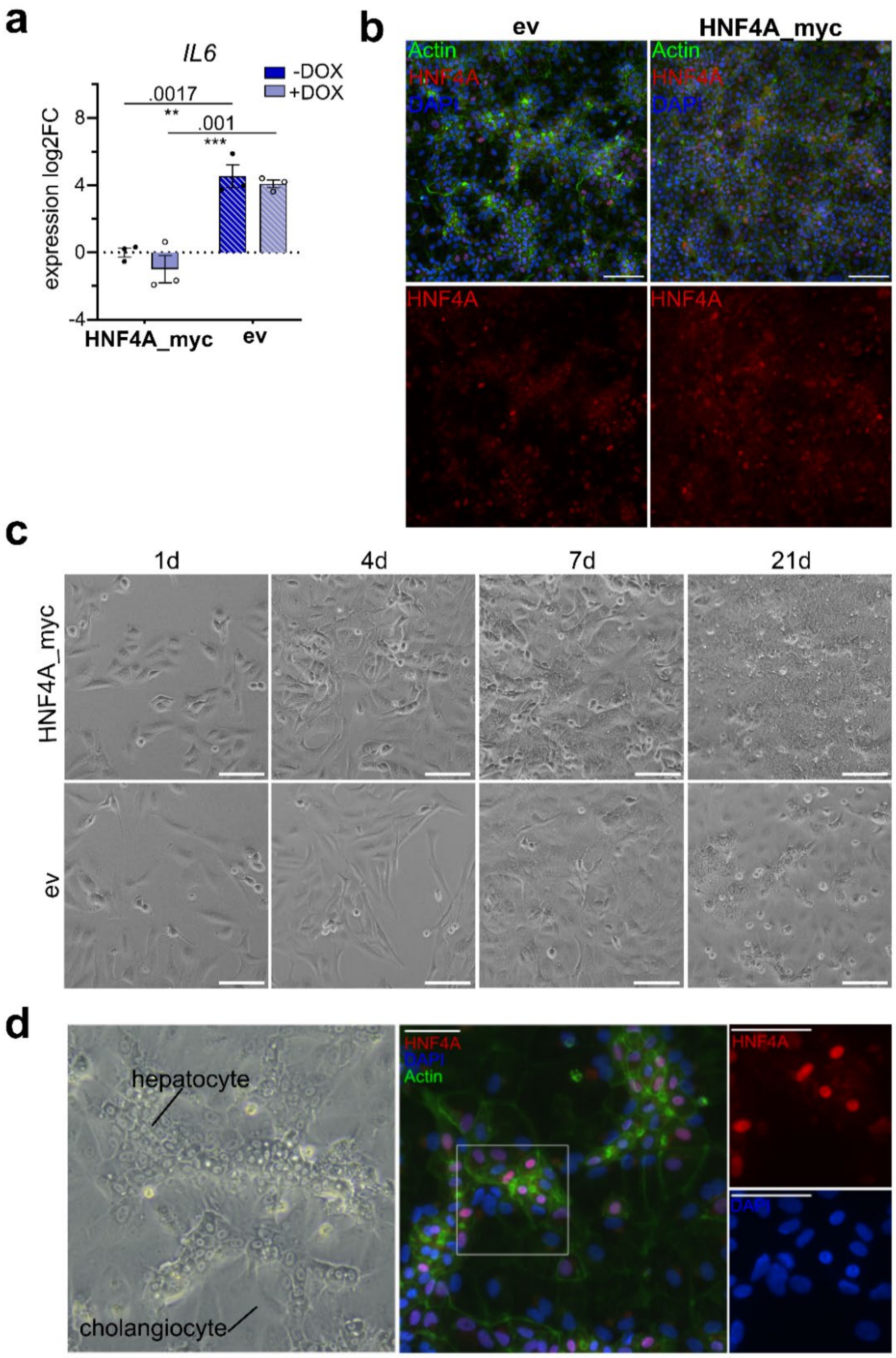

Fig. S6: dHepaRG HNF4A_myc cell line with reduced cholangiocyte background and IL6 expression. (a) IL6 expression in dHepaRG HNF4A_myc and ev cell line with DOX treatment (n = 3, data displayed as mean±SEM, one-way ANOVA with Holm-Sidak's multiple comparison testing). (b) Immunofluorescence of HNF4A in dHepaRG ev and HNF4A_myc cell line depicts higher ratios of hepatic HNF4A positive cells in HNF4A_myc cell line (scale bar = 100 µm). (c) Brightfield microscopy images of HNF4A_myc and ev HepaRG cell lines during growth (day 1-7) and differentiation phases (day 7-21), scale bar = 100 µm. (d) Phase contrast and immunofluorescence microscopy of HNF4A in dHepaRG WT cells shows that only hepatocyte-specified cells are HNF4A positive (scale bar = 100 µm).

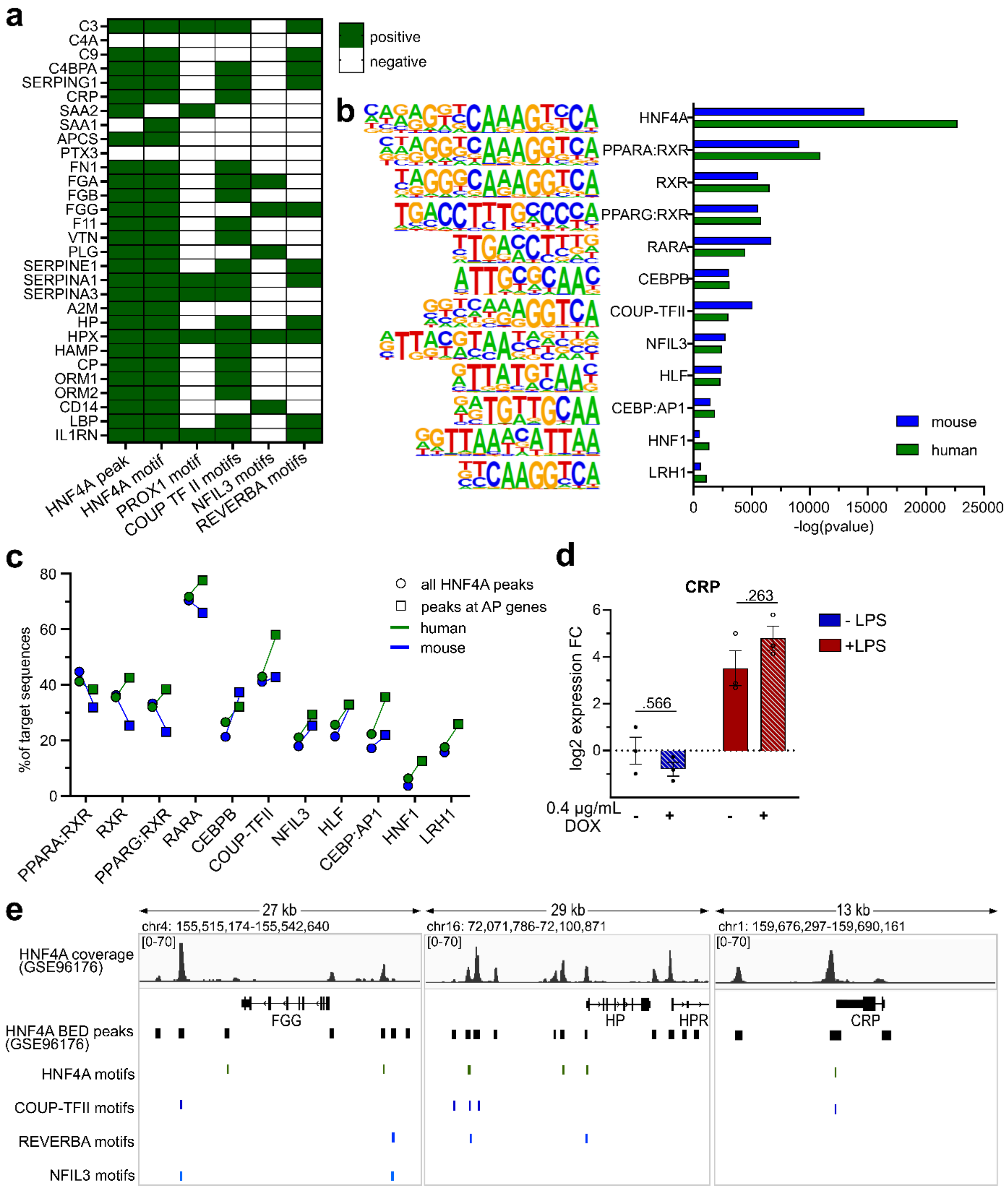

Fig. S7: Motif analysis of HNF4A binding sites proximal to AP genes.
(a) Occurrence of HNF4A ChIP peak and motifs for HNF4A, PROX1, COUP-TFII, NFIL3 and REVERBA at the 30 well-described AP genes. (b) Commonly enriched TF motifs among both human and mouse HNF4A binding sites across the entire genome, left: consensus sequence as position weight matrix, right: motifs ranked by p-value. (c) Percentage of target sequences harboring motifs for variousTFs within HNF4A binding sites across the whole genome (circle) of human (green) or mouse (blue) or specific at AP genes (square). (d) Expression of *CRP* in dHepaRG HNF4A_myc cells after 6 h LPS treatment (200 ng/mL) and previous DOX-induction (48 h, 400 ng/mL) (n=3, 2-way ANOVA with post-hoc Sidak's test). (e) IGV browser track of human HNF4A ChIP-Seq (GSE96176) coverage and called peaks in addition to distinct motifs for HNF4A, COUP TF II, REVERBA or NFIL3 at loci for *FGG*, *HP* and *CRP*.

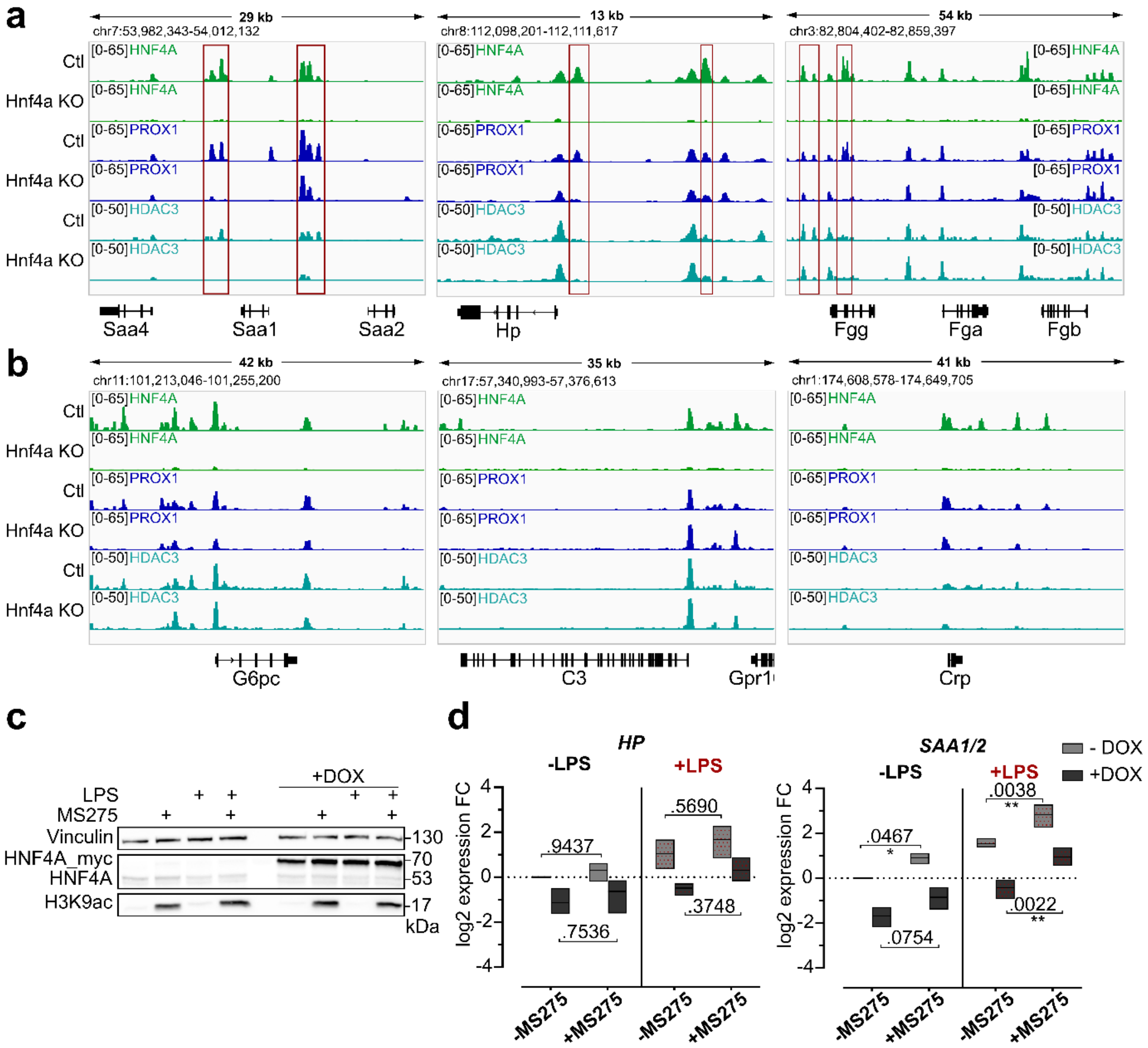

Fig. S8: HDAC-1/-3 inhibition ameliorates HNF4A-mediated repression of *HP* and *SAA*
(a, b) IGV browser tracks of HNF4A, PROX1 and HDAC3 ChIP-Seq in control and HNF4A KO mouse liver, data available from GSE90533, red boxes indicate sites where HNF4A knockout impacts PROX1/HDAC3 recruitment. (c) Whole cell extracts showing histone-hyperacetlytion after 6 h MS275-treatment in LPS/DOX-treated dHepaRG HNF4A_myc cells. (d) *HP* and *SAA1*/2 expression in dHepaRG HNF4A_myc cell line after 6 h of MS275 (1 µM) and LPS (200 ng/mL) co-treatment and previous DOX induction (48 h, 400 ng/mL) (3-way ANOVA post hoc Sidak's test). n = 3, box plot from min-max, line at mean.

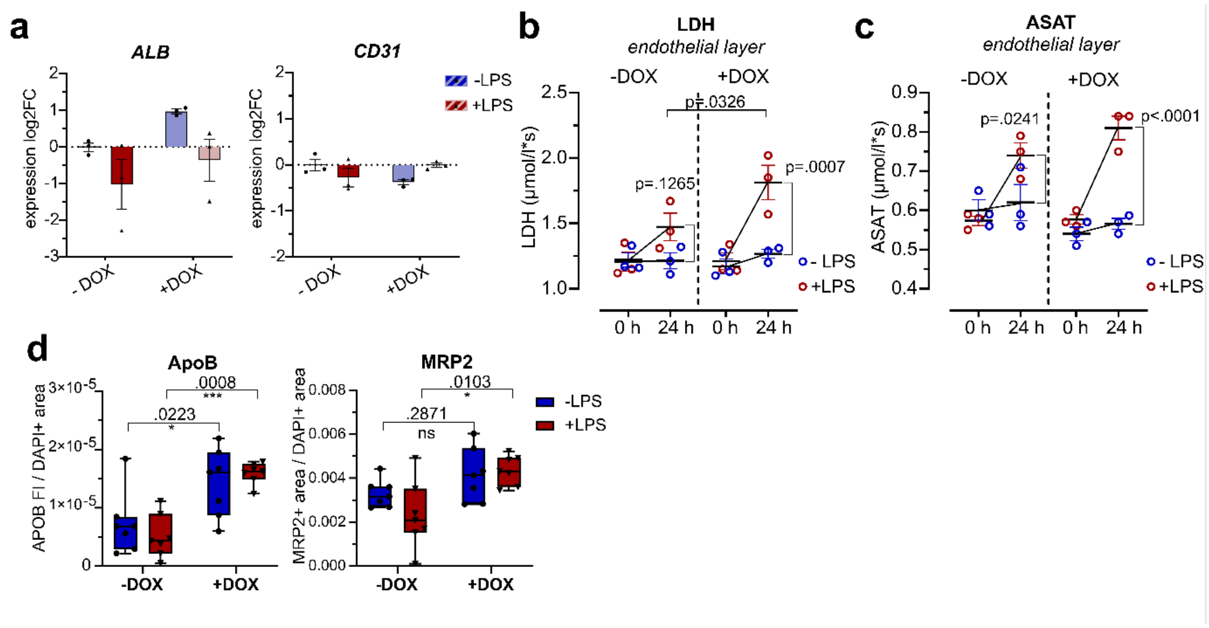

Fig. S9: Supplementary data for HNF4A overexpression in the Liverchip

(a) RT-qPCR showing expression levels albumin (ALB) and CD31 mRNA from whole liverchip RNA. (b) LDH and (c) ASAT concentrations in endothelial cavity. (b, d: 2-way ANOVA with Holm-Sidak's multiple comparison test). (d) quantification of ApoB Fluorescence intensity and MRP2 positive area over DAPI positive area, as determined with FIJI on at least 2 individual images each from 3 biological replicates. n = 3, data displayed as mean±SEM.

Fig. S10 (following page): Original uncropped Western blot images.

The molecular weight ladder used in each blot refers to: PageRuler Prestained Plus Protein Ladder (Thermo Fisher Scientific #26619). R1 (replicate 1) refers to image depicted in main figure, R2/3 are remaining two biological replicates not appearing in main figure. Loading orders are given from left to right.

(a) Figure 2a, loading order: control, IL1β, IL6, PMA, LPS, E. coli; 6 h left, 24 h right.

(b) Figure 2b (HepaRG), loading order: 0, 1, 3, 6, 9, 12 h LPS; R3 only right part of blot.

(c) Figure 2b (HepG2), loading order: 0, 1, 2, 3, 6, 9 h IL6/IL1β.

(d) Figure 2c, lanes 1-4: 0, 6, 15, 24 h CHX; lanes 5-7: 6, 15, 24 h CHX+IL6/IL1β; lane 8: IL6/IL1β.

(e) Figure 3a; lanes of interest for HepG2 ev are only the last two of each set (lane 5/12 0 h; lane 6/13 6 h). Blots labeled "CYT" have cytosolic fraction loaded on the left, and WCE on the right. Blots labeled "CHR" have nuclear soluble fraction loaded on the left, and chromatin on the right.

(f) Figure 4b (HepG2), loading order R1: 0, 25, 100, 400 ng/ml HNF4A_myc left, ev right; R2: 0, 100, 500 ng/ml HNF4A_myc on lanes 1-3, ev on lanes 4-6; R3: 0, 0, 100, 100, 500, 500 ng/ml HNF4A myc, ev 0, ev 100 ng/ml.

(g) Figure 4b (HepaRG): triplicates separated by ladder, loading order for each set: lane 1 ev, lanes 2-4: HNF4A_myc (25, 100, 400 ng/ml DOX).

(h) Figure S4c, loading order: 0, 1, 2, 3, 9, 24 h PMA.

(i) Figure S4d, lanes of interest are first three left from the protein ladder. Loading: 0, 1, 6 h LPS.

(j) Figure S5b, loading R1/2: lane 1-untransfected control, 2-control + cytokines, 3-siHNF4A, 4-siHNF4A+cytokines, 5-siNeg, 6-siNeg+cytokines. loading R3: 1-siNeg, 2-siNeg+cytokines, 3-siHNF4A, 4-siHNF4A+cytokines.

(k) Figure S8d, loading order: 1-control, 2-MS275, 3-LPS, 4-LPS+MS275; left: w/o DOX, right: with DOX.

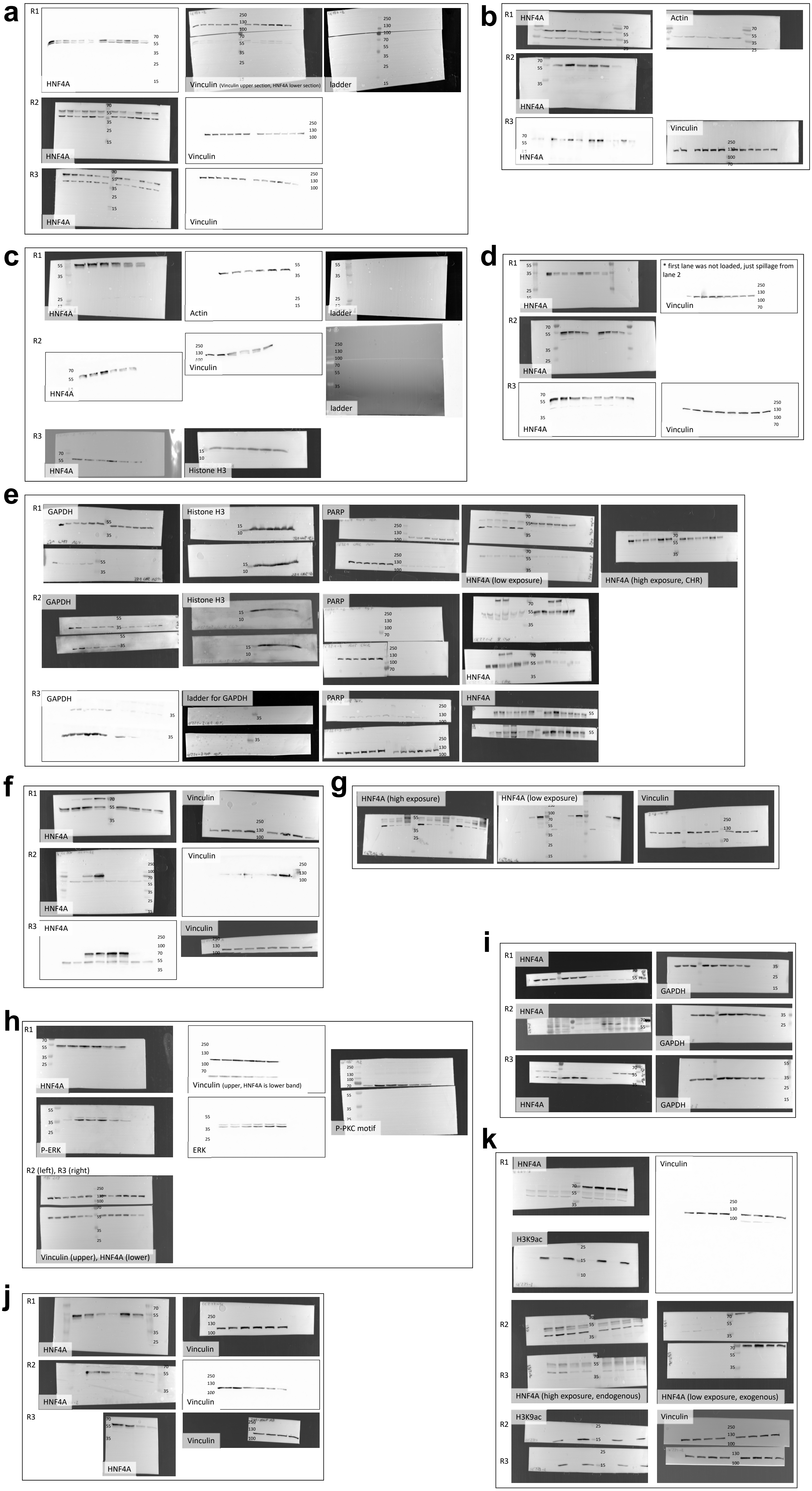
a
R1
HNF4A
Vinculin (Vinculin upper section, HNF4A lower section)
ladder
R2
HNF4A
Vinculin
R3
HNF4A
Vinculin
b
R1
HNF4A
Actin
R2
HNF4A
R3
HNF4A
Vinculin
c
R1
HNF4A
Actin
ladder
R2
HNF4A
Vinculin
ladder
R3
HNF4A
Histone H3
d
R1
HNF4A
* first lane was not loaded, just spillage from lane 2
Vinculin
R2
HNF4A
R3
HNF4A
Vinculin
e
R1
GAPDH
Histone H3
PARP
HNF4A (low exposure)
HNF4A (high exposure, CHR)
R2
GAPDH
Histone H3
PARP
HNF4A
R3
GAPDH
ladder for GAPDH
PARP
HNF4A
f
R1
HNF4A
Vinculin
R2
HNF4A
Vinculin
R3
HNF4A
Vinculin
g
HNF4A (high exposure)
HNF4A (low exposure)
Vinculin
h
R1
HNF4A
Vinculin (upper, HNF4A is lower band)
P-PKC motif
P-ERK
ERK
R2 (left), R3 (right)
Vinculin (upper), HNF4A (lower)
i
R1
HNF4A
GAPDH
R2
HNF4A
GAPDH
R3
HNF4A
GAPDH
j
R1
HNF4A
Vinculin
R2
HNF4A
Vinculin
R3
HNF4A
Vinculin
k
R1
HNF4A
Vinculin
H3K9ac
R2
R3
HNF4A (high exposure, endogenous)
HNF4A (low exposure, exogenous)
R2
H3K9ac
Vinculin
R3
