# Supplementary material for: Downregulation of HNF4A enables transcriptomic reprogramming during the hepatic acute-phase response
Source: Commun Biol. 2024 May 16;7:589. doi: 10.1038/s42003-024-06288-1 (PMC11099168; doi:10.1038/s42003-024-06288-1)
Supplement: Supplementary file 2 — Description of Additional Supplementary Files [file 42003_2024_6288_MOESM2_ESM.pdf]

# Description of Additional Supplementary Files

**File name:** Supplementary Data File 1

**Description:** FIMO motif analysis on HNF4A ChIP GSE96176.

**File name:** Supplementary Data File 2

**Description:** HOMER motif analysis on human and mouse HNF4A ChIP datasets (GSE96176,GSE90533).

**File name:** Supplementary Data File 3

**Description:** The source data behind the graphs in the paper
